# Preclinical Evaluation of Novel Sterically Optimized VLP-Based Vaccines against All Four DENV Serotypes

**DOI:** 10.3390/vaccines12080874

**Published:** 2024-07-31

**Authors:** Dominik A. Rothen, Sudip Kumar Dutta, Pascal S. Krenger, Anne-Cathrine S. Vogt, Ilva Lieknina, Jan M. Sobczak, Albert D. M. E. Osterhaus, Mona O. Mohsen, Monique Vogel, Byron Martina, Kaspars Tars, Martin F. Bachmann

**Affiliations:** 1 Department of BioMedical Research, University of Bern, 3008 Bern, Switzerland; martin.bachmann@me.com (M.F.B.); 2 Department of Immunology RIA, University Hospital Bern, 3010 Bern, Switzerland; 3 Graduate School of Cellular and Biomedical Sciences, University of Bern, 3012 Bern, Switzerland; 4 Artemis Bioservices, 2629 JD Delft, The Netherlands; 5 Latvian Biomedical Research & Study Centre, Ratsupites iela 1, LV 1067 Riga, Latvia; 6 Research Center for Emerging Infections and Zoonoses, University of Veterinary Medicine Hannover, 30559 Hannover, Germany; 7 Jenner Institute, Nuffield Department of Medicine, University of Oxford, Oxford OX3 7DQ, UK

**Keywords:** virus-like particles, dengue virus, vaccine

## Abstract

Over the past few decades, dengue fever has emerged as a significant global health threat, affecting tropical and moderate climate regions. Current vaccines have practical limitations, there is a strong need for safer, more effective options. This study introduces novel vaccine candidates covering all four dengue virus (DENV) serotypes using virus-like particles (VLPs), a proven vaccine platform. The dengue virus envelope protein domain III (EDIII), the primary target of DENV-neutralizing antibodies, was either genetically fused or chemically coupled to bacteriophage-derived AP205-VLPs. To facilitate the incorporation of the large EDIII domain, AP205 monomers were dimerized, resulting in sterically optimized VLPs with 90 N- and C-termini. These vaccines induced high-affinity/avidity antibody titers in mice, and confirmed their protective potential by neutralizing different DENV serotypes in vitro. Administration of a tetravalent vaccine induced high neutralizing titers against all four serotypes without producing enhancing antibodies, at least not against DENV2. In conclusion, the vaccine candidates, especially when administered in a combined fashion, exhibit intriguing properties for potential use in the field, and exploring the possibility of conducting a preclinical challenge model to verify protection would be a logical next step.

## 1. Introduction

Dengue fever, a mosquito-born viral infection, currently represents one of the world’s most important tropical diseases. As the distribution of the disease caused by the dengue viruses (DENV) is strongly linked to the distribution of the mosquito vectors *Aedes aegypti* and *Aedes albopictus*, it is widely spread throughout the tropics, in particular in crowded urban centers [1,2]. These regions include more than 129 countries [3], with Asia accounting for up to 70% of the disease’s global burden and being a hotspot of dengue infections [4]. For the year 2022, more than 4 million cases, including more than 4000 deaths, have been reported [5]. While most dengue infections are asymptomatic or exhibit only minor symptoms, they can also present as a serious, flu-like sickness that affects newborns, young children, and adults but rarely results in death unless infection causes dengue shock syndrome [6].

DENV is a positive-sense single-stranded RNA virus belonging to the genus *Orthoflavivirus* [7] of the *Flaviviridae* family. There are four serotypes of the dengue virus (DENV 1–4) and infection by any of them can lead to several different clinical manifestations, which are important for disease severity [8]. A 30% divergence in the DENV polyprotein between the four serotypes has been demonstrated, and several genotypes within each serotype show different geographical distributions [9].

The spectrum of the clinical illness caused by DENV can be classified into three stages: dengue fever (DF), dengue hemorrhagic fever (DHF), and dengue shock syndrome (DSS) [6]. Whereas DF is a self-limiting fever lasting up to 7 days, hemorrhagic manifestations and plasma leakage occur additionally in DHF [10]. When plasma loss reaches a critical stage, DSS ensues, and the patient may die within 12–24 h [11]. In a primary infection, high titers of IgM and IgG can be noticed at days 3–5 and 6–10, respectively, after the onset of the infection. IgG will persist and will deliver a life-long immunity, but only against the specific serotype. The first infection may or may not result in a symptomatic infection, whereas the secondary infection with a different DENV serotype more frequently results in classical DF [12]. During a second infection with a different serotype, the presence of low amounts of heterotypic antibodies, which are cross-reactive but only weakly neutralizing, can lead to an increase in viral load and severity of the disease in a process called antibody-dependent enhancement (ADE) [13].

DENV virions possess an icosahedral envelope organization and a spherical nucleocapsid core [14]. The mature virion consists of three structural proteins, the capsid protein C, the membrane protein M, and the envelope protein E. The capsid protein (11 kDa) encapsulates the RNA genome and forms the viral nucleocapsid [15,16]. The nucleocapsid is finally surrounded by a host-cell-derived lipid bilayer, which incorporates 180 copies of each M (8 kDa) and E proteins. The dengue E (55 kDa) protein is a large and cysteine-rich protein whose main function is binding to the host cell receptor, fusion, and entry into the host cells. The ectodomain consists of three structural domains. Domain I lies in between the homodimerization domain II and the immunoglobulin-like domain III [17]. Since the majority of neutralizing antibodies are directed against domain III, it is of great significance for vaccine development [18,19,20]. Through its major role in receptor binding for viral entry [21] and its recognition by most neutralizing antibodies [22,23], hence preventing the virus from entering its host cells and resulting in disease prevention, the EDIII presents the ideal target for vaccine strategies.

A currently approved vaccine is CYD-TDV, also called Dengvaxia, made by Sanofi Pasteur. It is a live attenuated tetravalent chimeric vaccine that is based on recombinant DNA technology, where the PrM and E structural genes of the yellow-fever-attenuated 17D strain vaccine were replaced with those of the four dengue serotypes. After a vaccination program run by the Philippine Department of Health, the manufacturer recommended that the vaccine should only be used in endemic regions and already previously infected, as in previously uninfected people, it might lead to a higher risk of severe cases of the disease likely due to ADE [24]. QDENGA^®^ (TAK-003), (Takeda Pharmaceutical, Tokyo, Japan) the second approved dengue vaccine, is a tetravalent vaccine based on the backbone of the live-attenuated serotype 2 virus [25]. The vaccine has been approved by the National Health Surveillance Agency (ANVISA) for use in DENV seronegative and seropositive individuals aged 4 to 60 [26]. In July 2023, Takeda announced a voluntary withdrawal of the vaccine in the U.S. on aspects of data collection [27]. Additionally, another live-attenuated dengue vaccine currently in phase 3, TV003 by Instituto Butantan, showed 89.5% and 69.6% efficacy against DENV-1 and DENV-2 [28,29]. However, no efficacy data for DENV-3 and DENV-4 are available since these serotypes did not circulate in the country during the study.

Although these new and large studies on tetravalent live-attenuated vaccines are promising, this specific vaccination approach suffers from several drawbacks. Since they use a live attenuated virus as a backbone, there remains a risk of reversion into a virulent strain, in particular as the molecular reasons for attenuation are poorly understood. Furthermore, due to this property, such vaccinations cannot be utilized in immunocompromised individuals, such as HIV patients, cancer patients undergoing chemotherapy or radiation treatment, or pregnant women. Another critical factor is that live attenuated vaccines require special handling during storage and transportation due to the requirement to retain them at low temperatures. Given that dengue is widespread in primarily tropical nations with poor infrastructure, this can be particularly challenging. Many attenuated dengue vaccines finally suffer from “viral interference” i.e., responses against some viral serotypes will dominate and suppress the other serotypes. Using a recombinant approach as done here, such problems may be less expected and could potentially be compensated by adjusting the dosage of vaccines for individual strains. For a dengue vaccine to be safe against primary and secondary infections, serotype-specific neutralizing antibodies, preferably in the absence of serotype cross-reactive (disease-enhancing) antibodies, may be the key, and only using one serotype is unlikely to reach that goal.

Virus-like particles (VLPs) are multi-protein supra-molecular structures, which carry many characteristics of viruses. They are considered traditional vaccine platforms and are used in several marketed vaccines against Hepatitis B virus (HBV), Hepatitis E virus (HEV), and Human papillomavirus (HPV) [30]. They possess many important traits, like allowing the display of complex and native antigens in a highly repetitive form on their surface through several methods such as chemical coupling or genetic fusion techniques. VLPs do not contain any replication-competent genetic material and are therefore also suitable for administration in immunocompromised people since they do not pose the risk of reversion to virulence [31]. In addition, VLPs are able to readily reach the draining lymph nodes due to their nanoparticle size. As a consequence, and most importantly, they have been shown to induce strong immune responses in all species tested [32,33,34,35].

The VLPs used here are based on the RNA bacteriophage AP205 infecting the Gram-negative bacteria *Acinetobacter* sp. AP205 VLP consists of 180 copies of monomer protein and is arranged in T = 3 geometry [33]. T = 3 VLPs constitute a promising vaccine geometry as they can cross-link 90–180 BCR on B cells, resulting in a stronger immune response [36]. In the present study, we used a recently developed form called AP205 dimer, consisting of 90 dimers of 2 fused monomers. This allows the incorporation of larger epitopes as the VLP has 90 rather than 180 N- and C-termini due to dimerization, where one N-terminus and one C-terminus were used to fuse two monomers. Due to the bacterial expression of AP205 VLPs, ssRNA, which serves as TLR 7/8 ligand, can be packaged and is seen to induce the most protective IgG subclasses in comparison to VLPs packed with other types of RNA [37] and the expression of inflammatory and type-1 IFN genes [38]. An important advantage of AP205 is the possibility to fuse both ends of the VLP-subunit resulting in a sterically optimized VLP allows the display of either N- or C-terminal epitopes of rather large size [39]. As AP205 was shown to self-assemble even when fused to a cysteine-containing epitope, it presents the ideal carrier for complex antigens [40] as the dengue virus envelope protein. Production of the vaccine in a bacterial host also enables a high yield at low cost and high speed, which facilitates potential use in less affluent countries [39].

In this report, we present newly developed dengue vaccines based on AP205 VLPs that incorporate the envelope protein domain III of each DENV serotype with chemical coupling or fusion techniques. The vaccines were evaluated for immunogenicity and safety in a murine model and were also tested for their ability to induce neutralizing antibodies against all four DENV serotypes. Additionally, the vaccines underwent testing for tetravalent use and were found to induce strong neutralizing antibodies against all four serotypes in the absence of disease-enhancing antibodies against the tested DENV-2.

## 2. Materials and Methods

### 2.1. Mice

Wild-type C57BL/6JOlaHsd mice were purchased from Envigo (Indianapolis, IN, USA). All in vivo experiments were conducted using 8–12-week-old female mice. Animal procedures adhered to the Swiss Animal Act (455.109.1—5 September 2008). Experimental protocols were approved by the Swiss Federal Veterinary Office

### 2.2. SDS-PAGE Analysis

Adapted from Krenger et al., 2023 [40]. After mixing 3 μL of reduction buffer (Thermo Scientific, Waltham, MA, USA, Cat. 39000) with 15 μL of the sample (1 mg/mL), the mixture was heated for 5 min at 95 °C and then loaded onto a 12% SDS-PAGE with a 4% stacking gel. After loading a 6 μL protein ladder (Thermo Scientific, 471 Cat. 26616), the gel was run at 70 V using the buffer specified as follows: Tris (hydroxymethyl)-aminoethane 2.5 mM, Glycine 25 mM, SDS 0.01%. After staining protein bands with InstantBlue^®^ Coomassie Protein Stain (Abcam, Cat. Ab119211, Cambridge, UK), an Azure Biosystems c300 was used to take a gel photograph with the visible channel set to auto-exposure time.

### 2.3. Agarose Gel Analysis

15 μL of the sample (1 mg/mL) was combined with 2.5 μL of loading dye (New England BioLabs, Cat. B7024S, Ipswich, MA, USA) and loaded onto a 2% Agarose (BioConcept, 7-01P02-R, Switzerland) gel using a ladder (Thermo Scientific, Cat. SM0242). The gel was then run in Tris-borate-EDTA (TBE) buffer at 50 V. The Azure Biosystems c300 was used to capture a gel image with the UV302 channel using auto-exposure time.

### 2.4. DV1-AP205, DV3-AP205, and AP205-DV4 Vaccine Cloning, Expression, and Production

**Figure 1:**
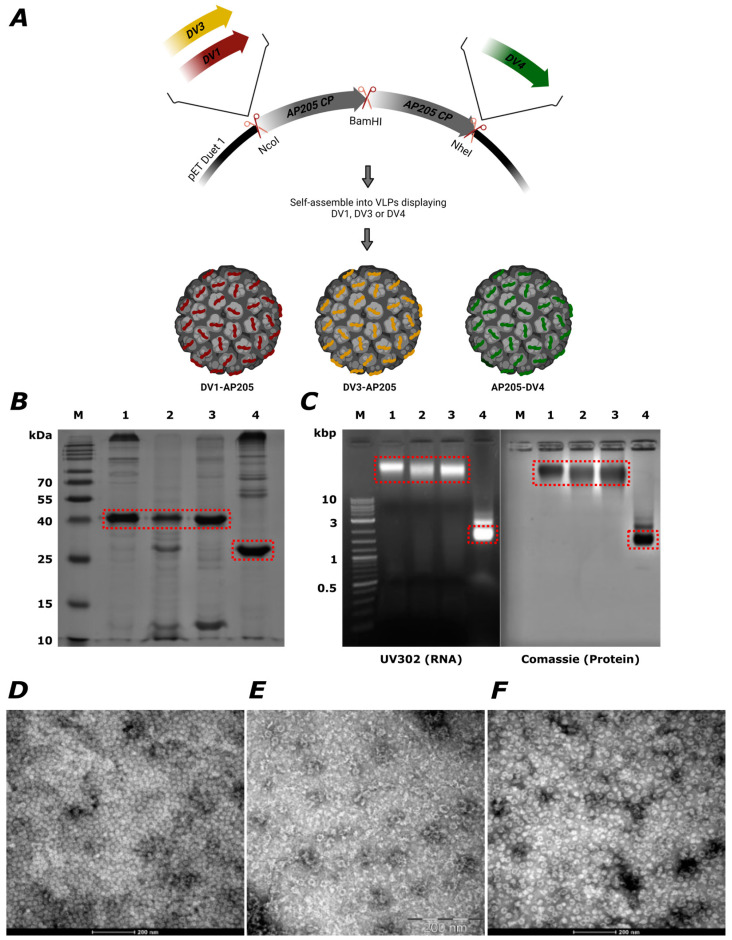
Effective integration of dengue virus envelope protein domain III (EDIII) into AP205 dimer VLPs generating a fusion vaccine. (**A**) Schematic illustration of the gene map for expression of the fusion products based on self-assembly of genetically modified AP205 dimer (consisting of two AP205 coat proteins (CP)) where the envelope protein domain III of dengue Serotype 1, 3, and 4 viruses (DV1, DV3, DV4) was N- or C-terminally fused to (**B**) 12% SDS-PAGE for DV1-AP205, DV3-AP205, and AP205-DV4 production. M. Protein marker, 1. DV1-AP205, 2. DV3-AP205, 3. AP205-DV4, 4. AP205 dimer. Products are indicated in the red boxes. Bands were visualized with InstantBlue^TM^ Comassie stain. (**C**) Agarose gel analysis was used to visualize the packed RNA in the fusion products and the correlating protein staining with Comassie. M. DNA Ladder, 1. DV1-AP205, 2. DV3-AP205, 3. AP205-DV4, 4. AP205 dimer. Products are indicated in the red boxes. (**D**) Electron microscopy (EM) of DV1-AP205. (**E**) EM of DV3-AP205. (**F**) EM of AP205-DV4. Scale bar 200 nm.

BL21 P812 *E. coli* bacteria were transformed with the pETDuet1 plasmid containing the AP205 dimer fused to the dengue virus envelope protein domain III of the individual serotypes 1, 3, or 4 (GenBank DV1: AEL79839.1, DV3: AAC63314.1, DV4: AGW23588.1). DV1 and DV3 were fused N-terminally to the AP205 dimer and DV4 C-terminally (Figure 1A). After transformation, several colonies were inoculated in Lysogeny broth medium and cultured overnight at 37 °C. 100 µg/mL Kanamycin was used for artificial selection. The next day, the culture was induced with 1mM IPTG after reaching an OD600nm between 0.4–0.6 and cultured overnight at 20 °C. The cells were harvested by centrifugation for 30 min at 10,000× *g* at 4 °C.

### 2.5. Protein Refolding and Purifying

To purify the wanted proteins from inclusion bodies, the pelleted cells were resuspended in 10 mL lysis buffer (PBS, 20 mM Tris, 5 mM EDTA, 5% Glycerol, 0.5% Triton X100, pH 8.0, 5 mM Betamercaptoethanol, 1:500 Lyzozyme) for 1 g cells. Then, cells were sonicated and centrifuged for 30 min at 10,000× *g* at 4 °C and the supernatant was discarded. The pellet was washed 4 times in lysis buffer. For the individual wash steps, the pellet was resuspended, sonicated, and centrifuged for 30 min at 10,000× *g* at 4 °C. After washing, the debris was resuspended in inclusion bodies (IB) solubilization buffer (8 M UREA, 50 mM Tris-HCl, 150 mM NaCl, pH 9.5) and incubated for 16 h at 4 °C on a rotating wheel with slow rotation. Next, the sample was centrifuged for 30 min at 10,000× *g* at 4 °C. The IB supernatant was then dialyzed against refolding buffer (RB) I buffer (2 M UREA, 100 mM Na_2_HPO_4_ × 2H_2_O, 100 mM NaH_2_PO_4_ × H_2_O, 0.5 M Arginine, 5 mM glutathione reduced, 0.5 mM glutathione oxidized, pH 9.5) for 24 h at 4 °C, and subsequently against RB II buffer (100 mM Na_2_HPO_4_ × 2H_2_O, 100 mM NaH_2_PO_4_ × H_2_O, 0.5 M Arginine, 5 mM glutathione reduced, 0.5 mM glutathione oxidized, pH 9.5) for 24–36 h at 4 °C, and finally against PBS at 4 °C. The dialyzed IB supernatant was centrifuged for 30 min at 10,000× *g* at 4 °C. The supernatant was further analyzed on SDS PAGE. The obtained proteins were purified on the appropriate gel filtration column, either Sepharose 4GG, Sepharose CL-4B, or Sephacryl S-500.

### 2.6. AP205~DV2 Vaccine Coupling and Production

SATA reagent was added to DV2 protein (GenBank: AGG79340.1) in accordance with the manufacturer’s instructions (Thermo Scientific, Rockford, IL, USA). This introduced a sulfhydryl group to the protein. A 6-fold molar excess of SATA was mixed with DV2 pro-tein, and the mixture was incubated for 30 min at room temperature (RT). A desalting column (Thermo Scientific, Rockford, IL, USA) was used to remove unreacted SATA. By combining the protein solution with 1/10 volume of the deacetylation solution (0.5 M hy-droxylamine, 25 mM EDTA in PBS, pH 7.2–7.5) and letting it sit for two hours at room temperature, free sulfhydryl groups were produced. A desalting column was used to eliminate hydroxylamine. The DV2 protein that had been sulfhydrylated was immediately employed for chemical coupling or AP205dimer VLPs. VLPs were incubated for 60 min at room temperature after being mixed with a 10-fold excess of SMPH crosslinker (Thermo Scientific, Rockford, IL, USA). With a desalting column, the remaining crosslinker was removed. The sulfhydryl-modified DV2 protein and the amine-modified VLPs were then mixed, and the mixture was incubated for an additional night at 4 °C. A portion of the unreacted DV2 protein was then eliminated by gel filtering on a Superdex200 10/300 GL column (GE Healthcare, Uppsala, Sweden) in PBS. Using SDS/PAGE, coupling efficiency was ascertained.

### 2.7. Electron Microscopy

The physical stability and integrity of the candidate vaccines were visualized by transmission electron microscopy (Philips CM12 EM, Eindhoven, The Netherlands). In order to image the grids, 10 μL of a pure vaccine (diluted to a concentration of 0.7–1 mg/mL) was added and left for 30 s. After 3-fold ddH2O washing, grids were negatively stained for 30 s with μL of 5% uranyl acetate. After pipetting away any extra uranyl acetate, the grids were allowed to air dry for 10 min. The magnification used to capture the images was 84,000× and 110,000×.

### 2.8. Vaccination Regimen

Wild-type C57BL/6JOlaHsd mice (8–12 weeks, Envigo) were vaccinated subcutaneously with 20 µg of either DV1-AP205, AP205~DV2, DV3-AP205, or AP205-DV4 vaccine in a volume of 100 µL or AP205dimer VLPs as a control in a volume of 100 µL without any adjuvants. The mice were boosted with an equal dose at day 28 and terminally bled at day 49. Serum was collected on a weekly basis via tail bleeding, and the serum was isolated using a microtainer tube (BD Biosciences, Franklin Lakes, NJ, USA).

For the follow-up experiment, wild-type C57BL/6JOlaHsd mice (8–12 weeks, Envigo) were also used. Group 1, consisting of 6 mice, was injected with a total of 20 µg (10 µg each) of DV1-AP205 and AP205-DV4 in a volume of 100 µL without any adjuvants. Group 2, also consisting of 6 mice, was injected with a total of 20 µg (5 µg each) of DV1-AP205, AP205~DV2, DV3-AP205, and AP205-DV4 in a volume of 100 µL without any adjuvants. On day 28, the mice received an identical dosage boost and were terminally bled on day 49. Weekly tail bleeding was used to collect serum, which was later separated using a mi-crotainer tube.

### 2.9. Enzyme-Linked Immunosorbent Assay (ELISA)

In order to calculate the total IgG antibody titers, 1 µg/mL of either recombinant DENV1 envelope protein domain III, DENV2 envelope protein domain III, DENV3 envelope protein domain III, or DENV4 envelope protein domain III (made in-house) diluted in PBS was coated onto ELISA Corning^TM^ 96-Well Half-Area Plates (Fisher Scientific, Hampton, NH, USA) in a volume of 50 µL/well. On a shaker, the plates were incubated at 4 °C for the entire night. The following day, PBS-tween 0.01% was used to wash the plates in the ELISA washer (Bio-Tek, 405 TS, Winooski, VT, USA). The plates were incubated at room temperature (RT) for two hours on a shaker after being blocked with 100 µL/well PBS-Casein 0.15%. The plates were flicked to get rid of the blocking solution. All serum samples were serially diluted at 1:20 and then at 1:3, with the exception of the final row, which served as a negative control. The plates were shaken at room temperature for two hours. Secondary anti-mouse IgG Fc gamma conjugated with Horse-Radish Peroxidase (Jackson Immunoresearch Cat. No. C840T69, West Grove, PA, USA) (1:1000) was added 50 µL/well to the plates after they had been rinsed with PBS-tween 0.01%. After another hour of incubation on a shaker, the plates were washed, developed, and an OD450 reading was taken (BioTek, Winooski, VT, USA). The reciprocal 50% dilution of the maximal OD450 was used to define OD50 values.

The same protocol was followed to evaluate the subclass antibody response, except using a different secondary antibody that was equivalent to the subclass: goat anti-mouse IgG2c (Southern BioTech, Cat. No 1078-05, 1:2000 dilution, Birmingham, AL, USA), goat anti-mouse IgG2b (Invitrogen, Waltham, MA, USA, Ref. M32407, 1:2000 dilution), and goat anti-mouse IgG3 (Southern BioTech, Cat. No 1101-05, 1:4000 dilution, AL, USA).

Goat anti-mouse IgA POX (ICN 55549, ID 91, 1:1000 dilution) was used as the secondary antibody, and the plates were coated with 1 µg/mL DENV envelope protein domain III in order to detect IgA antibodies. A further step was carried out to reduce the amount of IgG before the serum was incubated. Protein G beads (10 µL; Invitrogen, USA) were placed in a tube and then placed under a magnet. After draining the liquid, the beads were combined with 75.6 µL of diluted sera in PBS-Casein 0.15%. For ten minutes, the tube was incubated at room temperature using a rotator. The sera were added to the first row of the ELISA plate, and the tubes were reinserted into the magnet. And then the ELISA was carried out, as previously mentioned.

### 2.10. Avidity (ELISA)

Two sets of plates were prepared in order to measure the avidity of the IgG antibodies. One set of plates was washed three times for five minutes with 50 µL/well 7M Urea in PBS + 0.05% Tween20, while the other set was washed with the same quantity of PBS + 0.05% Tween20. Both sets were coated with 1 µg/mL DENV envelope protein domain III. Every plate was cleaned using the ELISA washer and PBS + 0.01% Tween 20 in between cycles. The remaining steps are the same as those of a typical ELISA, as previously mentioned.

### 2.11. Cell Culture

C6/36 (CLR-1660) mosquito cell line (American Type Culture Collection (ATCC)) was cultured in Eagle’s Minimum Essential Medium (EMEM) (Lonza, Basel, Switzerland) supplemented with 10% heat-inactivated fetal bovine serum ((HIFBS) (Lonza Benelux BV, Breda, The Netherlands), 0.75% sodium bicarbonate (NaHCO_3_) (Lonza), 10 mM HEPES buffer (Lonza) and 1% penicillin, streptomycin (Pen-Strep) (Lonza)) at 28 °C in an incubator without CO2. Vero cells (ATCC^®^ CCL-81™, Manassas, VA, USA) cultured in complete media (composition: Dulbecco’s modified Eagle medium (DMEM) with 10% HI-FBS (Lonza Benelux BV, Breda, The Netherlands), supplemented with 0.75% NaHCO_3_, 10 mM HEPES buffer (Lonza), and 1% penicillin, streptomycin (Pen-Strep) (Lonza)) at 37 °C in a humified incubator with 5% CO_2_. SC (CLR-3622) cells, obtained from ATCC and cultured with complete media containing: RPMI 1640 supplemented with 50 µM beta-mercaptoethanol, penicillin (100 U/mL, Lonza), streptomycin (100 µg/mL, Lonza), and 10% HI-FBS (Gibco/ThermoFisher, Paisley, UK) at 37 °C in 5% CO_2_ in a humidified incubator. All three cell lines were routinely tested negative for mycoplasma using an in-house developed RT-PCR primer and probes [41].

### 2.12. Virus

Dengue serotypes 1 (VR1856, Hawaii); 2 (VR1584, New Guinea C) and 3 (VR1265, H87 [V-576-001-022]) (ATCC^®^, Manassas, VA, USA) were first amplified in C6/36 cells. The resulting supernatant containing the virus was then used to infect Vero cells at a multiplicity of infection (MOI) of 0.01. After 72 h, the virus was harvested. Dengue serotype 4 (VR-1490, H241 (TC adapted) (ATCC^®^) was directly amplified in Vero cells at an MOI of 0.01, and the viruses were harvested based on the observed cytopathic effect. The harvested virus stocks were clarified by centrifugation and stored at −80 °C.

The titers of dengue serotypes 1–4 were determined by incubating 10-fold serial dilutions of virus stock on Vero cells for 4 days at 37 °C with 5% CO_2_. An in-house immunostaining technique was utilized to count the number of virus-infected cells. In summary, 2.5% formalin was used to fix the infected cells, and 0.1% Triton-X-100 was used to permeabilize them in 70% ethanol. Rabbit anti-flavivirus group antigen monoclonal antibody (Absolute antibody, Oxford, UK) was used to stain the infected cells, and Goat anti-rabbit IgG (H + L) Highly Cross-Adsorbed Secondary Antibody, Alexa Fluor Plus 488 (2 mg), from Invitrogen, was used to detect the infection. The nucleus was then counterstained in the wells using 4′,6-Diamidino-2-Phenylindole, Dihydrochloride (DAPI) from Thermo Fisher Scientific. Plates were scanned at a 4× objective with a Cytation1V Imaging Reader (BioTek), and the Gen5 software v2.1 (BioTek) was used for analysis. The virus stock titer was calculated using the Karber formula [42].

### 2.13. Focus Reduction Neutralization Test (FRNT)

The neutralization capacity of antibodies produced after immunizing mice with either DV1-AP205, AP205~DV2, DV3-AP205, AP205-DV4 was determined using FRNT assay. Human convalescent serum 001 to dengue Virus (NR-50226) was used as a positive control, while sera from wild type C57BL/6JOlaHsd mice was used as a negative control. In brief, a 96-well substrate plate was prepared the day before the experiment using Vero cells (2 × 10^4^ cells/well) grown in full medium. To inactivate the complement, the serum from vaccinated mice was heat-inactivated at 56 °C for 30 min. It was then serially di-luted two times in infection media (DMEM supplemented with 0.75% NaHCO_3_, 10 mM HEPES buffer, 1% Pen-Strep, and 1% HI-FBS), ranging from 1:20 to 1:160. Each well was then filled to the same volume with 500TCID50 of various dengue serotypes, and the mixture was incubated for an hour at 37 °C. After adding the viral serum mixture to the substrate plate, it was incubated in 5% CO_2_ at 37 °C for 60 min. Subsequently, the monolayer underwent a single PBS wash, after which the infection media was added. It was then incubated for 48 h at 37 °C with 5% CO_2_. To assess the percentage of infected cells, an in-house-designed immunofluorescent test (Supplementary Figure S6) was employed, as detailed above. The fifty percent focus reduction neutralization test (FRNT50) titer of each sample was determined by comparing the number of infected cells to the virus control. Each serum sample was tested in duplicate.

### 2.14. Antibody-Dependent Enhancement Test (ADE) with Dengue Virus (DENV-2/NGC Strain)

To determine whether the vaccinated mice had sera-induced ADE against DENV-2, SC (CLR-3622, ATCC) cells were used. The ADE assay was conducted after stimulating/differentiating SC cells (5 × 10^5^ cells/mL) with Concanavalin A (1:1000) (Merck, Darmstadt, Germany) and Croton oil (1:20,000) (Merck) for 72 h. Once the mature macrophage-like stage was reached, the stimulation media was removed, and the cells were washed with PBS and maintained in complete media (described above) for 48 h.

Afterward, serial two-fold dilutions of the sera from vaccinated mice were prepared, ranging from 1:20 to 1:160, and the dengue virus type 2/NGC strain was added at a multiplicity of infection (MOI) 0.1. The mouse anti-Flavivirus envelope protein IgG2a monoclonal antibody (4G2) (AbFLAVENV-4G2-200) was used as a positive control, and serum from wild-type C57BL/6OlaHsd mice was used as a negative control. The mixture was incubated for 1 h at 37 °C and subsequently incubated with mature macrophage-like cells. After an incubation period of 2 h, the mixture was removed, washed with PBS, and further incubated in a fresh medium for another 48 h. After 48 h, RNA was isolated from the cells containing the supernatant, followed by a pan DENV real-time polymerase chain reaction (RT-PCR) as described previously [43]. To quantify the fold change in viral RNA level, the Ct-value of each sample was compared to the virus control. Each serum sample was tested in duplicate.

### 2.15. Statistical Analysis

Data were analyzed and presented as mean ± SEM using an unpaired Student’s *t* test or one-way ANOVA with Tukey correction for multiple comparisons, as mentioned in the figure legend, using GraphPad PRISM 9.4 (GraphPad Software Inc., San Diego, CA, USA). The value of *p* < 0.05 was considered statistically significant (* *p* < 0.05, ** *p* < 0.01, *** *p* < 0.001, **** *p* < 0.0001).

## 3. Results

### 3.1. Effective Integration of Dengue Virus Envelope Protein Domain III into AP205 Dimer VLPs Generating a Fusion Vaccine

To develop a new vaccine candidate against the respective DENV serotypes, the sterically optimized AP205 VLP platform was used. AP205 VLPs consist of 180 copies of coat protein and are arranged in a T = 3 symmetrical configuration [33]. This platform allows the genetic fusion of peptide epitopes to be displayed on their surface and it is able to elicit potent immune responses. The VLPs are very receptive to accepting foreign sequences up to 55 amino acids and have the ability to accept epitopes at both ends of the VLP-subunit, allowing the display of either N- or C-terminal epitopes [44]. By using sterically optimized VLPs based on AP205 dimers, epitopes may be larger, as this particle only has 90 rather than 180 N- and C-termini [39]. The VLPs can be produced recombinantly in *E. coli* bacteria [39], presenting the possibility of upscaling production. A schematic illustration of the gene map for expression of the fusion vaccines is displayed in Figure 1A. The constructs are based on fusing the envelope protein domain III of dengue Serotype 1, 3, and 4 viruses (DV1, DV3, DV4) either N- or C-terminally to the AP205 dimer, which consists of two AP205 genetically fused coat proteins (CP). It has been shown in previous studies that the domain III of the envelope protein induces antibodies capable of completely neutralizing the virus [18,45] and is therefore an optimal target for dengue vaccine development [46]. The VLPs were successfully produced through the expression of the plasmids in *E. coli* bacteria, refolded, and purified. The refolding process presents a unique technique by which the coat proteins of the VLPs undergo reassembly within an UREA gradient, facilitating the reconstitution of proteins from inclusion bodies. The VLP protein of interest is around 39 kDa in size (AP205 dimer; 28 kDa, DENV envelope protein domain III (DVs); 11 kDa) (Figure 1B). The production of the proteins was confirmed by SDS-PAGE, showing the bands for the fusion products at the expected size of about 39 kDa in comparison to the AP205 dimer without EDIII fusion at around 28 kDa.

Re-assembly of the VLPs in the presence of RNA co-purified with the VLP-subunits, allows for the spontaneous packaging of ssRNA, which serves as a toll-like receptor (TLR) 3,7/8 agonist, promoting the activation of antigen-presenting (APCs) B cells [40,47] and is seen to induce the most protective IgG subclasses and maintain the high avidity and diversity of antibodies [37]. The packaging of ssRNA was verified by agarose gel analysis (Figure 1C), where it could be seen that the produced VLPs included ssRNA at the same band height. To finally confirm the integrity and folding of the particles, electron microscopy (EM) analysis was performed, demonstrating that the VLPs of 25–30 nm in size exhibited no evidence of aggregation or structure alteration (Figure 1D,E). For unknown reasons, the biochemical properties of DENV-2 EDIII expression failed in the generation of VLPs, despite several attempts by varying the size of the fused domain and the fusion site. Therefore, the alternative of chemical coupling was chosen to display the protein of interest on the VLPs’ surface (see below).

### 3.2. AP205 Dimer Is an Effective Platform for Developing a Vaccine against Dengue Serotype 2 via Chemical Coupling

**Figure 2:**
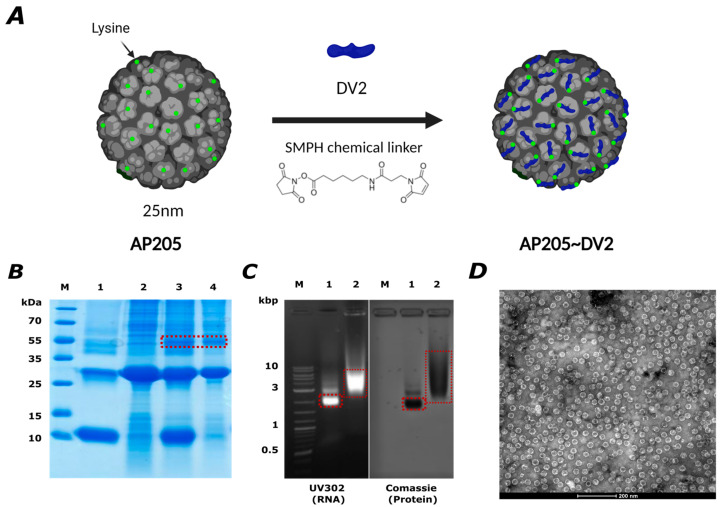
The AP205 dimer is an effective platform for developing a vaccine against dengue serotype 2 via chemical coupling. (**A**) Schematic representation of the chemical coupling of the dengue serotype 2 virus envelope protein domain III (DV2) to the AP205 dimer via the SMPH bifunctional cross-linker. (**B**) 12% SDS-PAGE for AP205~DV2 production. M. Marker, 1. DV2, 2. AP205 dimer with SMPH, 3. AP205~DV2 before gel filtration, 4. AP205~DV2 after gel filtration. Red box indicates the coupled product. Bands were visualized with InstantBlue^TM^ Comassie stain. (**C**) Agarose gel analysis to visualize the packed RNA in the coupled product and the correlated protein staining with Comassie. M. DNA Ladder, 1. AP205 dimer, 2. AP205~DV2. Products are indicated in the red boxes. (**D**) Electron microscopy (EM) of AP205~DV2. Scale bar 200 nm.

Because fusion of the DENV-2 EDIII (DV2) to AP205 failed, the protein was chemically coupled to the VLPs’ surface using a SMPH hetero-bifunctional cross-linker as a classical alternative [48]. The schematic representation of the chemical coupling is displayed in Figure 2A. The coupling is verified by SDS-PAGE, where the coupling product with a size of around 39 kDa can be detected in between the 35 kDa and 55 kDa marker bands (Figure 2B). The samples that only contain DV2 and the AP205 dimer show no band at this position. Through the expression of the unconjugated AP205 dimer in bacteria, the coupled product is self-adjuvanted with prokaryotic ssRNA packaged during self-assembly in bacteria, which is confirmed in an Agarose gel showing the packed RNA at the same position as the protein in the gel (Figure 2C). EM was used to examine the integrity of the VLPs after the coupling process, and no evidence of aggregation was found (Figure 2D).

### 3.3. Vaccination with the Newly Developed Vaccine Candidates Induces a Strong Humoral Immune Response

**Figure 3:**
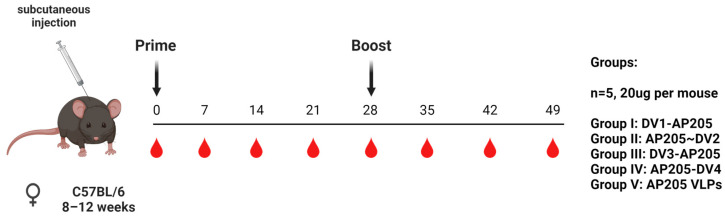
Vaccination overview. Vaccination regimen (prime on day 0 and boost on day 28, 20 µg per mouse, subcutaneous injection), bleeding time points, and groups. Figure created with Biorender.com.

**Figure 4:**
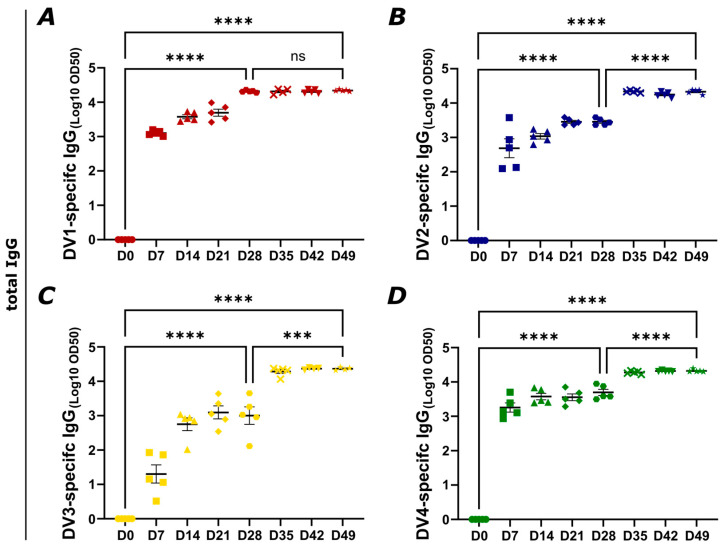
Vaccination with the newly developed vaccine candidates induces a strong humoral immune response. (**A**) DV1-specific IgG titer induced by DV1-AP205. (**B**) DV2-specific IgG titer induced by AP205~DV2. (**C**) DV3-specific IgG titer induced by DV3-AP205. (**D**) DV4-specific IgG titer induced by AP205-DV4. Days 0, 7, 14, 21, 28, 35, 42, and 49 are shown. Titer was measured by ELISA, LOG10 OD50 as shown. Statistical analysis (mean ± SEM) using one-way ANOVA. Vaccine groups n = 5. One representative of three similar experiments is shown. The value of *p* < 0.05 was considered statistically significant (ns *p* > 0.05, *** *p* < 0.001, **** *p* < 0.0001).

By employing dengue virus envelope protein domain III proteins (DV) of the various serotypes (1–4), the immunogenicity and the induced humoral immune responses in a murine model following vaccination with DV1-AP205, AP205~DV2, DV3-AP205, and AP205-DV4 were assessed. C57BL/6JOlaHsd mice were primed and boosted subcutaneously with 20 µg AP205 dimer VLPs as a control or with the vaccines (Figure 3). Because of the packaged RNA, no further adjuvant was used. Total DV-specific IgG was measured by ELISA on recombinant EDIII protein. Antibodies recognizing DV1 were already detected on day 7 post priming in the vaccine group with a high Log10 OD50 titer of around 3. The antibody titer peaked on day 28 and did not significantly increase after the second dosage, demonstrating a potent immunological response even after just one dose of the vaccine (Figure 4A). Similarly, the antibody response to DV2 could be seen within a week, and it gradually increased until boosting on day 28. The titer then peaked at similar levels as the DV1 response one week after the boost (Figure 4B). DV3-specific IgG antibodies were also detected on day 7 but with a lower titer in comparison to the DV1 and DV2 responses. The IgG titer increased over the following days, reaching its peak on day 35, one week after the boost (Figure 4C). The DV4-specific IgG response was already showing a high antibody titer one week post-priming. The response increased one week after the boost and reached its maximum on day 49 (Figure 4D). Sera from mice vaccinated with AP205 dimer VLPs as a control did not show any increase in DV-specific antibody titers (Supplementary Figure S1). Overall, a strong systemic and similar humoral immune response was induced upon vaccination, in particular after the boost.

### 3.4. Immunization with All Four Vaccine Candidates Induces IgG2c- and IgG2b-Dominated IgG Responses and Also Promotes Isotype Switching to IgA

**Figure 5:**
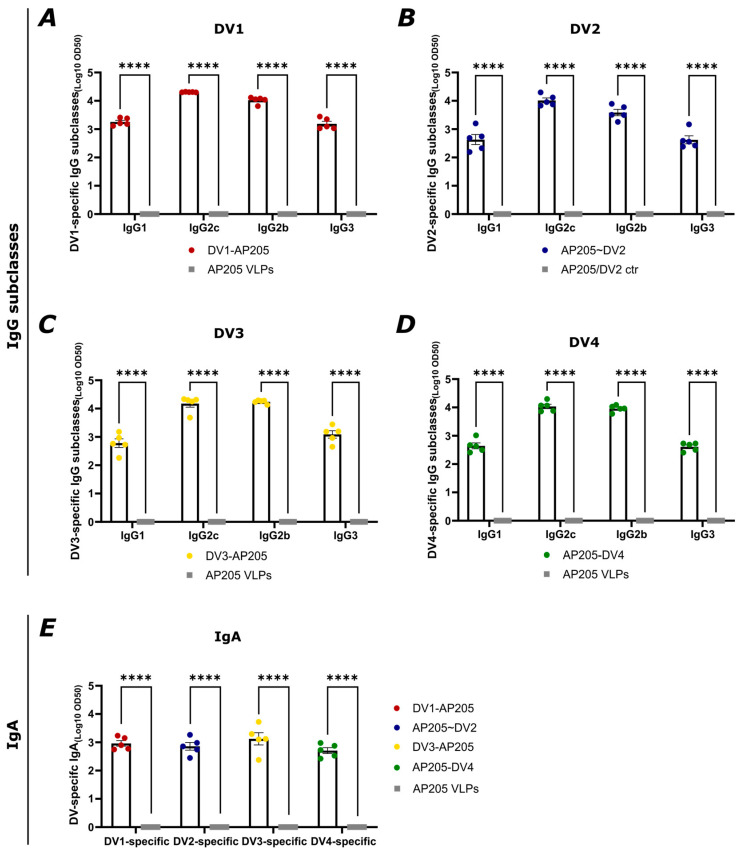
Immunization with all four vaccine candidates induces IgG2c- and IgG2b-dominant dengue virus envelope protein domain III-specific IgG subclass response and promotes isotype switching to IgA. (**A**–**E**) DV-specific IgG subclasses titer of day 42 from mice vaccinated with AP205 VLPs as the control and (**A**) DV1-AP205, (**B**) AP205~DV2, (**C**) DV3-AP205, and (**D**) AP205-DV4. (**E**) DV-specific IgA titer. Sera from day 42 was used. Titer was measured by ELISA, LOG10 OD50 as shown. Statistical analysis (mean ± SEM) using Student’s *t*-test. Control group n = 5, vaccine groups n = 5. One representative of two similar experiments is shown. The value of *p* < 0.05 was considered statistically significant (**** *p* < 0.0001).

IgG subclasses play a significant role in the immunological response to viruses because they modulate opsonization and other immune effector functions [49]. Furthermore, research has shown that DENV-specific IgA produced in response to DENV infection may regulate the overall IgG-mediated ADE activity and could serve as an indicator of reduced disease risk [50]. The ability of the here-produced DENV vaccines to produce IgG subclasses and IgA was assessed by running ELISA against the different EDIII domains (DV) of the envelope proteins from the DENV serotypes proteins with serum from day 42. The results showed an IgG2c and IgG2b dominating response amongst the subclasses for all vaccines (Figure 5A–D). Also, potent IgG1 and IgG3 induction can be seen, although lower than IgG2c and IgG2b. Isotype switching to IgA was also significantly enhanced in all vaccine groups, as shown in Figure 5E. The induction was balanced for all groups, with the DV3-specific IgA titer being the highest. The correlating OD450 values can be found in Supplementary Figure S2.

### 3.5. Vaccination with AP205-DV Vaccines Induces High-Avidity Antibodies Cross-Reactive to Other Dengue Serotype EDIII Domains

**Figure 6:**
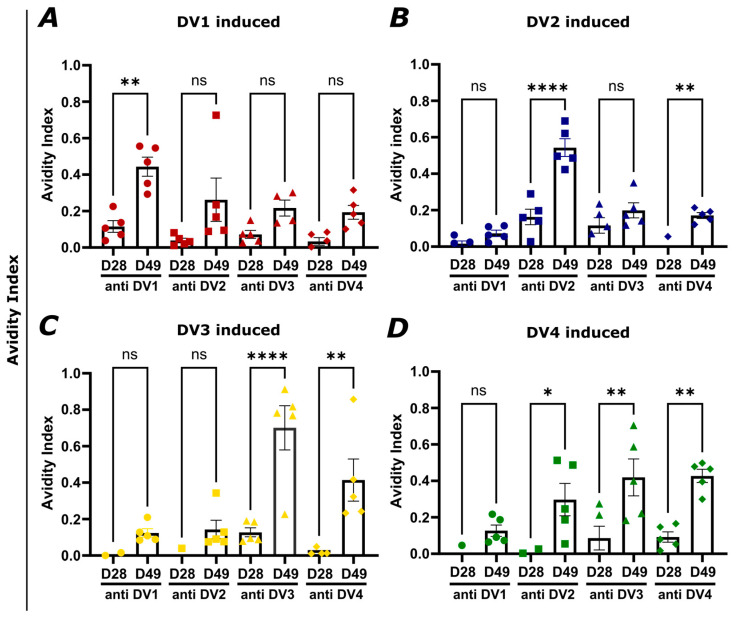
Vaccination with AP205-DV vaccines induces high-avidity antibodies cross-reactive to other dengue serotype EDIII domains. (**A**) Avidity Index (AI) of DV-specific IgG titer of the group vaccinated with DV1-AP205. (**B**) AI of DV-specific induced IgG titer of the group vaccinated with AP205~DV2. (**C**) AI of DV-specific induced IgG titer of the group vaccinated with DV3-AP205. (**D**) AI of DV-specific induced IgG titer of the group vaccinated with AP205-DV4. Sera used from day 28 and 49. Statistical analysis (mean ± SEM) using one-way ANOVA. Vaccine groups n = 5. One representative of two similar experiments is shown. The value of *p* < 0.05 was considered statistically significant (ns *p* > 0.05, * *p* < 0.05, ** *p* < 0.01, **** *p* < 0.0001).

It was demonstrated that IgG serum avidity to DENV gradually shifts from primarily focusing on probably low-affinity cross-reactive serotypes to the serotype of the current infection. More importantly, serum avidity correlates with neutralization capacity [51]. To determine whether the induced IgG antibodies against the different DV proteins are able to cross-react with other serotypes, and also to evaluate how strongly they can bind, avidity ELISAs were performed where plates were coated with the DV proteins, always in duplicates, and then either treated with PBST or with 7M UREA to eliminate low avidity antibodies. The presented avidity index in Figure 6 indicates the percentage of high avidity antibodies out of all IgG antibodies and is calculated by dividing the EC50 (optical density reading at 50% of the maximum signal) dilution of the urea-treated sample by the EC50 dilution of the control sample. The correlating Log10 OD50 values can be found in Supplementary Figure S3. In Figure 6A, the sera from the DV1-AP205 vaccinated group were tested for avidity binding to the four DV proteins. As expected, the binding avidity was the highest for DV1. Nevertheless, the cross-reactivity of the antibodies was noticeable through recognition of the other serotypes, but with a smaller avidity index. In Figure 6B, the sera from the AP205~DV2 vaccine group was tested. Also, here, against the correlating DV2 protein, the binding avidity was highest, with a very high value above 0.5 at day 49. Hence, over 50% of antibodies induced are of high avidity. The response against DV1 was the lowest, followed by DV4 and DV3. As shown in Figure 6C, the sera from the DV3-AP205 vaccine group were assessed for their antibody-binding avidity. Also, in this group, the response was the strongest against the inductive serotype, with a very high avidity index for DV3. Against DV4, the DV3-AP205 vaccine seemed to induce high avidity antibodies with a percentage of around 40% at day 49. The cross reaction against DV1 and DV2 was low, with values of around 0.1. Finally, as shown in Figure 6D, the AP05-DV4 induced sera were tested and showed a similar amount of high avidity antibodies, against DV3 and DV4, with values around 40%. About 30% of induced antibodies were of high avidity against DV2, followed by DV1 with around 10%, indicating a stronger binding to DV2 than to DV1. In general, the avidity was significantly higher for the specific DV protein after the boost on day 28. Overall, these results show that the engineered vaccines can induce high avidity antibodies against the DV of the correlating serotypes and are also able to cross-bind and recognize DVs from the other serotypes, but not as efficiently as the serotype that originally induced the antibodies.

### 3.6. Vaccination with All Four Vaccines Compared to Vaccination with Only Two (DV1-AP205/AP205-DV4) Increases the Humoral Immune Response with Higher Avidity

**Figure 7:**
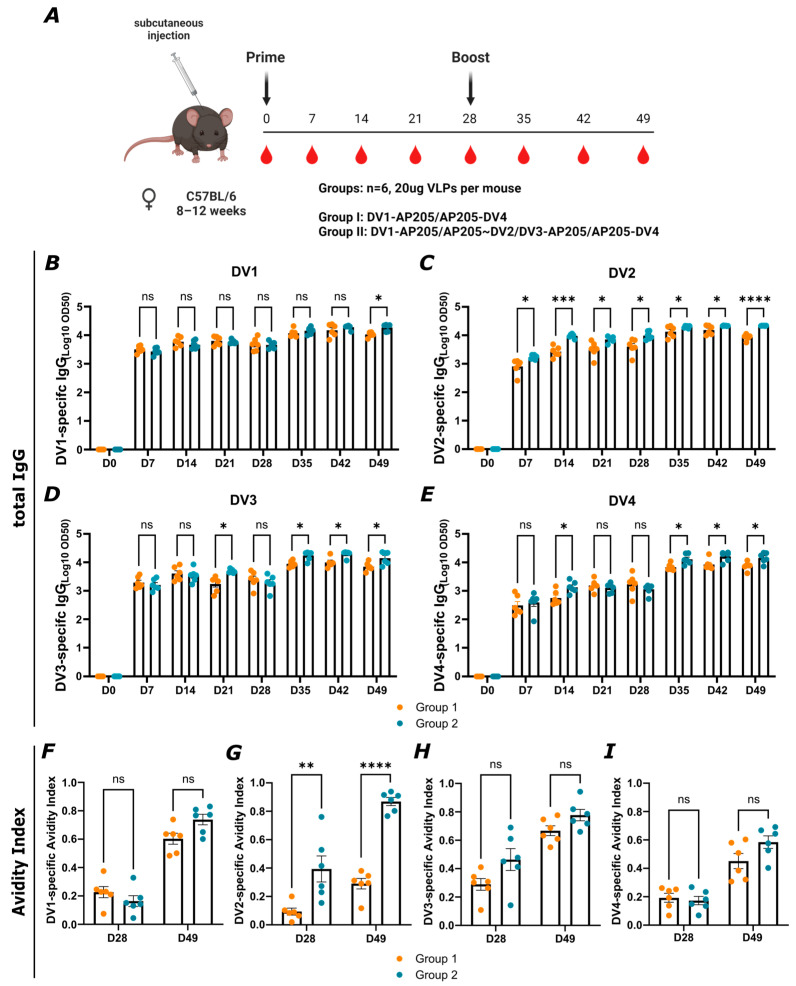
Vaccination with all four vaccines compared to vaccination with only two (DV1-AP205/AP205-DV4) tends to increase the humoral immune response with higher avidity. (**A**) Vaccination regimen (prime on day 0 and boost on day 28, 20 µg of total VLPs per mouse, subcutaneous injection), bleeding time points, and groups. Figure created with Biorender.com. (**B**–**E**) DV-specific IgG titer on days 0, 7, 14, 21, 28, 35, 42, and 49 from group 1 and group 2 measured by ELISA, LOG10 OD50 as shown. (**F**–**I**) DV-specific Avidity Index of group 1 and 2 from day 28 and day 49. Statistical analysis (mean ± SEM) using Student’s *t*-test. Group 1 n = 6, group 2 n = 6. One representative of two similar experiments is shown. The value of *p* < 0.05 was considered statistically significant (ns *p* > 0.05, * *p* < 0.05, ** *p* < 0.01, *** *p* < 0.001, **** *p* < 0.0001).

To assess bivalence compared to tetravalent vaccination, a group of mice (group 1) was vaccinated with DV1-AP205/AP205-DV4 and a second group (group 2) with all four vaccines, as illustrated in Figure 7A. The collected immune sera of both groups were tested for the total IgG response against the different dengue EDIII proteins. In Figure 7B, the DV1-specific response was assessed. The titer increased over time, reaching its peak on day 35, seven days after the boost. The two groups did not show a significant difference until day 49. The DV2-specific response showed an increasing titer from day 7 to day 28. After the boost on day 28, the titer reached its peak. Group 2 elicited a significantly higher IgG titer than group 1 against those strains not included in group 1 (Figure 7C). Figure 7D,E present the DV3- and DV4-specific IgG titers. For both, the highest titer could be seen after the boost. Figure 7F–I show the avidity index. On day 28, pre boost, the DV-specific avidity was always lower than on day 49, post boost, for both groups. Comparing group 1 and group 2, the difference was non-significant, except for the DV2-specific avidity index, which was higher in the tetravalent group. In general, group 2 tended to elicit higher avidity IgG abs than group 1, in particular against the strains not included in groups 1 (i.e., DV2&3). The avidity for group 2 after one boost was similarly high for the DV1-, DV2-, and DV3-specific responses, followed by a DV4-specific response, which was slightly lower. For group 1, the DV1- and DV3-specific avidities were highest, followed by the DV4- and DV2-specific responses.

### 3.7. The Individual Vaccines Are Able to Induce Neutralizing Antibodies

**Figure 8:**
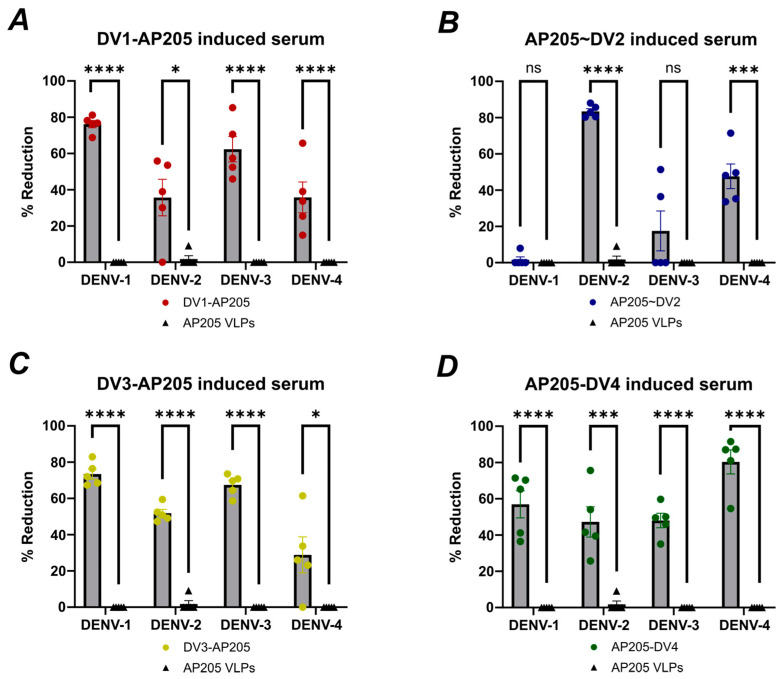
The individual vaccines are able to induce neutralizing antibodies. (**A**) Reduction of DENV-1-4 infection by DV1-AP205-induced serum (red points) and AP205 VLPs as the control (black triangles). Serum was diluted 1/100. (**B**) Reduction of DENV-1-4 infection by AP205~DV2 induced serum (blue points) and AP205 VLPs as the control (black triangles). Serum was diluted 1/100. (**C**) Reduction of DENV-1-4 infection by DV3-AP205-induced serum (yellow points) and AP205 VLPs as the control (black triangles). Serum was diluted 1/100. (**D**) Reduction of DENV-1-4 infection by AP205-DV4-induced serum (green points) and AP205 VLPs as the control (black triangles). Serum was diluted 1/100. Serum from day 49 was used. Vaccine groups n = 5, control group n = 5. One representative of two similar experiments is shown. Statistical analysis (mean ± SEM) using Student’s *t*-test. The value of *p* < 0.05 was considered statistically significant (ns *p* > 0.05, * *p* < 0.05, *** *p* < 0.001, **** *p* < 0.0001).

A Virus Neutralization Test (VNT) was performed to assess if the antibodies induced by the different DENV vaccines were able to neutralize DENV serotypes. Vaccination with DV1-AP205 elicits antibodies that exhibit significant efficacy in reducing DENV-1 infection by nearly 80%, while neutralization of the other strains was expectedly lower (Figure 8A). AP205~DV2-induced antibodies reduced DENV-2 infection efficiently compared to the other strains (Figure 8B). DV3-AP205-induced serum could reduce DENV-3 infection and DENV-1 infection similarly significantly, whereas for DENV-2 and DENV-4 infection, the reduction was lower (Figure 8C). Vaccination with AP205-DV4 reduced DENV-4 infection by 80%. (Figure 8D). The control serum did not show any significant reduction and stayed at 0% in all assays (Figure 8A–D).

### 3.8. Tetravalent Vaccination against DENV Induces Antibodies Able to Neutralize All Four DENV Serotypes

**Figure 9:**
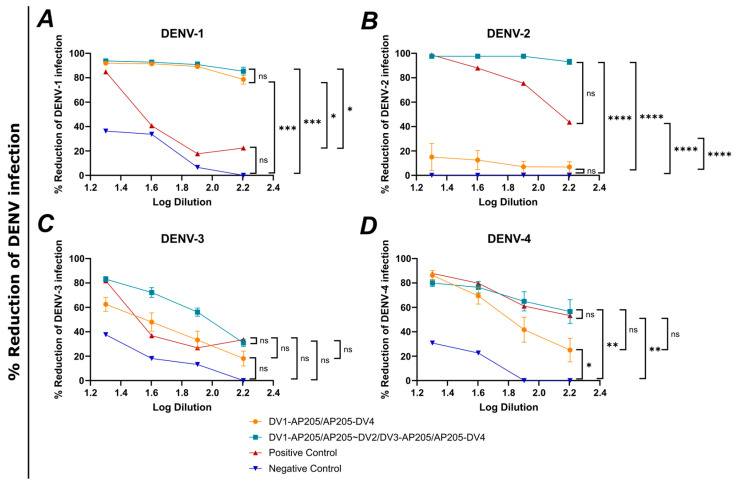

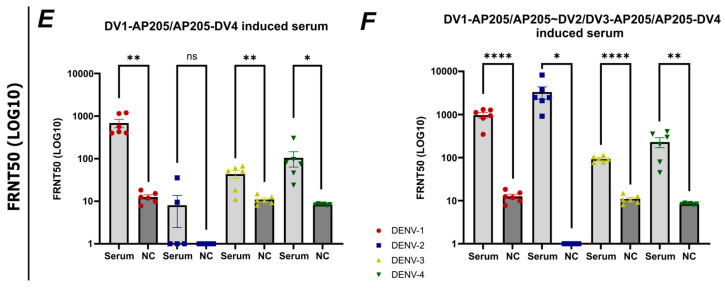
Tetravalent vaccination against DENV induces antibodies able to neutralize all four DENV serotypes. (**A**–**E**) Reduction of (**A**) DENV-1, (**B**) DENV-2, (**C**) DENV-3, (**D**) DENV-4 infection by DV1-AP205/AP205-DV4 induced serum (orange), DV1-AP205/AP205~DV2/DV3-AP205/AP205-DV4 induced serum (turquoise), Positive Control (red) and Negative Control (NC, blue). Serum dilution is shown in log values. Serum used from day 49. Data presented as a mean of 6 sera. (**E**) FRNT50-(Focus Reduction Neutralization Test 50) values from DV1-AP205/AP205-DV4 induced serum and Negative controls. DENV-1 neutralization is depicted with red points, DENV-2 with blue squares, DENV-3 with yellow upside triangles and DENV-4 with green downside triangles. (**F**) FRNT50-values from DV1-AP205/AP205~DV2/DV3-AP205/AP205-DV4 induced serum and Negative controls. DENV-1 neutralization is depicted with red points, DENV-2 with blue squares, DENV-3 with yellow upside triangles and DENV-4 with green downside triangles. Serum used from day 49. Naïve serum used as negative control. ATCC serum used as positive control. Statistical analysis (mean ± SEM) using Student’s *t*-test for (**E**,**F**) and one way ANOVA for (**A**–**D**). Vaccine groups n = 6. One representative of 2 similar experiments is shown. The value of *p* < 0.05 was considered statistically significant (ns *p* > 0.05, * *p* < 0.05, ** *p* < 0.01, *** *p* < 0.001, **** *p* < 0.0001).

As shown in Figure 9, groups that received two or four vaccines were assessed for their ability to neutralize all four DENV serotypes. As shown in Figure 9A–D, the percentage reductions of DENV infection are shown for the groups vaccinated with DV1-AP205/AP205-DV4 (bivalent) and DV1-AP205/AP205~DV2/DV3-AP205/AP205-DV4 (tetravalent). As shown in Figure 9A, the two vaccine groups reduced DENV-1 infection very similarly, with a non-significant difference. The reduction at the lowest dilution was for both groups at around 90%, going down toward 80%, with a higher dilution. Both groups showed a significant difference between the positive and negative controls, whereas the positive control reduced the DENV-1 infection by over 80% at the lowest dilution and dropped to around 20% with a higher dilution. The negative control showed a reduction of around 37% and dropped to 0%. As shown in Figure 9B, the group vaccinated with the tetravalent vaccine showed a very stable curve, and the reduction of DENV-2 infection stayed for every dilution at around 100%. The group administrated with bivalent vaccine had a significantly lower reduction of under 20% at the lowest serum dilution. The negative control had a 0% reduction at all dilutions, and the positive control started at 100% and dropped to around 50% the higher the dilution. The reduction of DENV-3 infection for the tetravalent group, as shown in Figure 9C, was higher than that for the bivalent group, although not significantly. While the tetravalent vaccine induced a reduction of around 80%, the bivalent vaccine only showed a 60% reduction at the lowest dilution. The negative control had the lowest reduction, with 40% at the lowest dilution, whereas the positive control reduced DENV-3 infection by around 80% at the lowest dilution. As shown in Figure 9D, both groups and the positive control showed a reduction of DENV-4 infection between 80 and 85%. The tetravalent group and the positive control dropped to around 60% at the highest dilution, whereas the bivalent group dropped to around 35%. Statistically, there was a non-significant difference among these groups. The negative control was significantly lower. The focus reduction neutralization test 50 (FNT50)-value is displayed in Figure 9E,F. For the group vaccinated with DV1-AP205/AP205-DV4 in Figure 9E, the FRNT50 for DENV-1 is around 690, significantly higher than for the negative control. The FRNT50 for DENV-2 is not significantly higher than the negative control, with a value of around 8. For DENV-3, the FRNT50-value is around 43, and for DENV-4, it is around 104. In Figure 9F, serum induced by tetravalent vaccination with DV1-AP205/AP205~DV2/DV3-AP205/AP205-DV4 reached a FRNT50-value for DENV-1 of around 966, for DENV-2 around 3329, for DENV-3 around 93, and for DENV-4 around 229, all values being significantly higher compared to the negative controls.

### 3.9. Antibodies Induced by Vaccination with DV1-AP205/AP205-DV4 Do Not Enhance DENV-2 Infection

**Figure 10:**
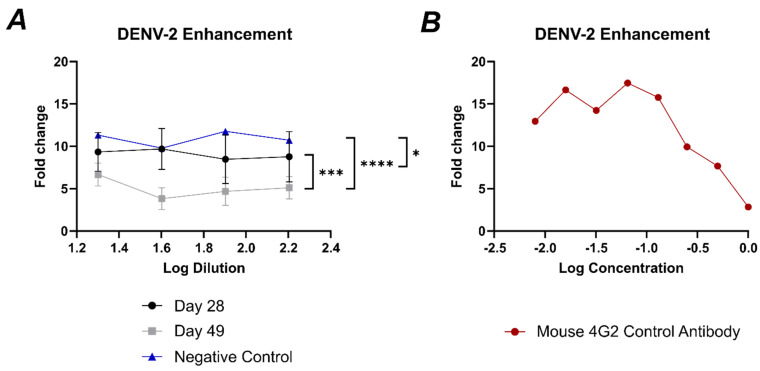
Antibodies induced by vaccination with DV1-AP205/AP205-DV4 do not enhance DENV−2 infection. (**A**) Fold change of DENV-2 infection of the serum-induced by vaccination with DV1-AP205/AP205-DV4. Day 28 is shown in black, and day 49 is shown in grey. Negative Control (naïve serum) is shown in blue. The serum dilution is shown in log values. (**B**) Fold change of DENV-2 infection of mouse 4G2 antibody used as a positive control. Antibody concentration is shown in log values. Statistical analysis (mean ± SEM) using one-way ANOVA. Vaccine group n = 6. One representative of two similar experiments is shown. The value of *p* < 0.05 was considered statistically significant (* *p* < 0.05, *** *p* < 0.001, **** *p* < 0.0001).

For assessing the potential disease enhancement properties of the induced antibodies, a DENV-2 enhancement assay was performed with the serum of the group vaccinated with DV1-AP205/AP205-DV4 (Supplementary Figure S4). In Figure 10A, the fold change of DENV-2 enhancement for the Negative Control and Day 28 and 49 of the induced serum is displayed. The Negative Control shows a 10–12-fold change of DENV-2 infection for the different serum dilutions. The serum of day 28 is significantly lower (*) than the Negative Control with a fold change between 8–10. Serum from day 49 is significantly lower than day 28 (***) and the Negative Control (****), with a 5-fold change in DENV-2 infection. For the Positive Control, a mouse 4G2 antibody was used at different concentrations and showed a 17.5-fold change in DENV-2 infection at an amount of 0.065 µg. Thus, our immune sera showed an ability to enhance infection that was lower than the negative control.

## 4. Discussion

Over the past decades, dengue has become an increasingly important global health threat in tropical regions. Due to rising urbanization, limited clean water supplies, and environmental change, which bring people and disease-carrying mosquito vectors, *Aedes aegypti* and *Aedes albopictus*, into closer contact, the disease has high potential for spreading more globally [52]. In response to the growing threat of dengue, vaccine research has gained prominence.

In this study, we present new vaccine candidates based on virus-like particles incorporating the EDIII of the different DENV serotypes. Unlike the vaccination candidates described earlier, the VLP vaccine only targets the EDIII and does not incorporate PrM, which could possibly induce undesirable, non-neutralizing, potentially enhancing abs. Furthermore, since the vaccine platform is stable at 4 °C over a long period of time, as shown in Supplementary Figure S5, storing and transport in many countries can be facilitated.

The immunogenic potential of our vaccine candidates was tested in a murine model, and the sera were analyzed in terms of the humoral immune response. The immunogenicity of an efficient vaccine is based on its ability to induce an antigen-specific B and T cell response [53]. Because substantial microbial replication is required in vivo for an efficient T cell response, which, due to immunizing with a non-replicating vaccine, is not provided, it is more challenging to induce a long-term T cell response [54]. Therefore, the main focus of a vaccine relies on the humoral immune response mediated by antibody production in B cells [53]. It has been shown that serotype-specific antibodies correlate with protection against DENV infection [55]. We could show that our newly developed vaccines are able to elicit a strong specific IgG response in mice, indicating that the humoral immune response was efficiently triggered.

DENV infection mainly induces IgG1 and IgG3 subclasses upon infection in humans [56]. In a murine model from a previous study, it was shown that bacterial RNA packed into VLPs can trigger TLR 3/7/8 in B cells, and induce subclass switching to IgG2c and IgG2b [37,40]. In a viral challenge, the IgG2 antibodies were of much higher importance in protection against mortality and morbidity because the Fc-γ regions of the IgG subclasses have unique effector interaction capabilities [57]. We could show in our study that, for all vaccine candidates, the IgG2 subclass titer was more prominent than IgG1/3. This isotype switching property of the vaccines may contribute their shown strong neutralizing capability but needs to be considered for potential disease enhancement due to a possible stronger Fc-receptor mediated viral uptake [58].

It has been demonstrated that DENV-specific IgA antibodies can effectively neutralize DENV but do not facilitate the ADE of DENV infection in vitro [50]. However, high-serum IgA titers have been linked to severe illness after secondary DENV infection, according to other research [56,59,60,61]. Nevertheless, the IgG-mediated ADE activity of DENV-immune plasma may be controlled overall by DENV-specific IgA in vivo; hence, IgA may also operate as a predictor of disease risk and high rations of IgA/IgG correlate with low disease burden [50]. Our data show significant induction of DENV-specific IgA immune responses upon vaccination, which is in agreement with earlier findings where it was shown that RNA-loaded VLPs also induce IgA responses [62]. This promising feature could be of great importance in inducing protection without disease enhancement.

Due to the high genomic similarity of up to 65% of the genome between different DENV serotypes [63], which are also similar to the E protein [64], cross-reactive antibodies are produced when vaccinated against or infected with different serotypes. It is important to note that cross-reactive antibodies may bind but not neutralize the virus, and therefore bear a risk for disease enhancement [65]. In general, serotype-specific nAbs are preferable to cross-reactive abs, since they bind more specifically to the virus and neutralize more efficiently [66]. With a tetravalent dengue vaccine, it is barely possible to prevent cross-reaction, since all four DENV serotypes are presented as targets to the immune system, including many conserved epitopes. Broad neutralizing antibodies may contribute to cross-neutralization [67]. In previous research, it could be found that the neutralization capacity of the serum may be correlated with serum avidity and antibody concentration, and higher avidity against heterologous serotypes may also imply protection [51]. By performing avidity ELISAs, the cross-reactivity and avidity of total IgG were assessed in our study, and we could show that all the vaccines induce cross-reactive IgG antibodies. However, the avidity was always highest against the serotype used for immunization. Interestingly, avidity strongly increased after a booster injection. This was also reflected in the VNT assays, where the neutralization potency of the individual vaccines was assessed for all four DENV serotypes. The best protection was always against the inductive serotype. Cross-neutralization was detectable, but only partly and not as potent compared to the inductive serotype. Nevertheless, the vaccines were shown to induce an immune response capable of efficiently neutralizing the correlating DENV serotype.

To design a tetravalent product, all four engineered VLPs were co-formulated in equal concentrations to obtain the final vaccine, which was then tested in mice (group 2). To compare the characteristics of the induced immune response, another group was added (group 1), where only the VLPs against DENV-1 and DENV-4 were co-formulated and injected. This choice is due to the fact that we wanted to assess for possible cross-recognition and cross-neutralization; thus, it makes sense to choose the most genetically diverse serotypes. DENV-1 is phylogenetically closest to DENV-3 and might be cross-protective, whereas DENV-4 is the most distant [68]. The total IgG responses of the groups were similarly high; in general, group 2 elicited a slightly higher antibody titer. Importantly, both groups induced a similar response as the individual vaccines, but only the tetravalent vaccine was able to elicit a balanced response against all four serotypes. In addition, the tetravalent vaccine also induced the highest avidity antibodies. This is an important finding, as avidity has been shown to correlate with neutralization [51].

Indeed, the tetravalent vaccine also induced the most pronounced and balanced neutralizing antibody response against all four serotypes. When comparing the tetravalent vaccine to the bivalent, it was evident that the protection and the correlating neutralization titer against DENV-2 and DENV-3, the two serotypes not covered in the bivalent mix, were notably lower. This underscores the critical importance of including all four DENV vaccines to elicit a well-rounded immune response.

Despite the good results for the tetravalent vaccine, it is essential to verify that there is no ADE effect induced upon vaccination. To this end, an ADE assay was conducted for DENV-2 enhancement. DENV-2 was selected as the serotype for this assay because, according to multiple studies, it has historically been more common, clinically significant, and linked to more severe cases of dengue sickness in dengue-endemic areas. To further confirm that the cross-reactive antibodies induced by the individual vaccines do not enhance DENV-2 infection, we utilized the serum from the group vaccinated with the bivalent vaccine, DV1-AP205/AP205-DV4. This vaccine formulation lacks the DENV-2 component, allowing us to ascertain whether the induced antibodies are capable of enhancing infection or not. This approach provides valuable insight into the specific role of antibodies elicited by the individual vaccines in modulating DENV-2 infection. Upon examination of the results, it was evident that no significant enhancement above negative serum was observed in the tested serum samples on either day 28 or day 49, even though enhancement was even lower after the boost. This finding provides additional support for the safety profile of the vaccine and confirms the specificity of the induced antibodies. These results offer reassurance regarding the vaccine’s ability to elicit a protective immune response without the risk of enhancing infection.

## 5. Conclusions

Within this research, we designed new, sterically optimized virus-like particles specific for the four DENV serotypes. The envelope protein domain III from DENV-1, DENV-3, and DENV-4 were genetically fused, and the DENV-2 EDIII was chemically coupled to the AP205 dimer VLP. The individual immune response of these new vaccine candidates was assessed for their induction of specific IgG antibodies, IgG subclasses, and IgA, and finally their potential to neutralize the correlating DENV serotype. The four VLPs were co-formulated as a new tetravalent vaccine candidate and further tested for immunogenicity. The VLPs were able to individually induce a strong and specific humoral immune response and neutralize the virus in a neutralization assay. Co-formulated, the vaccine was able to induce neutralizing antibodies against all four DENV serotypes without significant enhancement of DENV-2 replication. As a conclusion, this promising outcome underscores the potential of our newly developed tetravalent vaccine as a comprehensive and effective alternative in the ongoing pursuit of combating DENV infections.

## Data Availability

All data are available in the main text or the Supplementary Materials.

## Supplementary Materials

The following supporting information can be downloaded at: https://www.mdpi.com/article/10.3390/vaccines12080874/s1, Supplementary Figure S1: Vaccination with the newly developed vaccine candidates induces a strong humoral immune response; Supplementary Figure S2: Immunization with all four vaccine candidates induces IgG2c and IgG2b dominant dengue virus envelope protein domain III specific IgG subclass response and promotes isotype switching to IgA; Supplementary Figure S3: Vaccination with DV1-AP205, AP205~DV2, DV3-AP205, and AP205-DV4 elicits high avidity antibodies, which are able to recognize the other dengue serotype EDIII domains due to cross-reactive properties; Supplementary Figure S4: Vaccination with all four vaccines compared to vaccination with only two (DV1-AP205/AP205-DV4) tends to increase the humoral immune response with higher avidity; Supplementary Figure S5: Stability of DV1-AP205 after 6 months storage at 4 °C; Supplementary Figure S6: Fluorescence Microscopy images of controls on Cells for the Neutralization Assays.
