# Correction: Rothen et al. Preclinical Evaluation of Novel Sterically Optimized VLP-Based Vaccines against All Four DENV Serotypes. *Vaccines* 2024, *12*, 874

**DOI:** 10.3390/vaccines12101189

**Published:** 2024-10-18

**Authors:** Dominik A. Rothen, Sudip Kumar Dutta, Pascal S. Krenger, Anne-Cathrine S. Vogt, Ilva Lieknina, Jan M. Sobczak, Albert D. M. E. Osterhaus, Mona O. Mohsen, Monique Vogel, Byron Martina, Kaspars Tars, Martin F. Bachmann

**Affiliations:** 1 Department of BioMedical Research, University of Bern, 3008 Bern, Switzerland; 2 Department of Immunology RIA, University Hospital Bern, 3010 Bern, Switzerland; 3 Graduate School of Cellular and Biomedical Sciences, University of Bern, 3012 Bern, Switzerland; 4 Artemis Bioservices, 2629 JD Delft, The Netherlands; 5 Latvian Biomedical Research & Study Centre, Ratsupites iela 1, LV 1067 Riga, Latvia; 6 Research Center for Emerging Infections and Zoonoses, University of Veterinary Medicine Hannover, 30559 Hannover, Germany; 7 Jenner Institute, Nuffield Department of Medicine, University of Oxford, Oxford OX3 7DQ, UK

The authors would like to make the following corrections to this published paper [1].

In the original manuscript, Figure 10A contained an error in the labeling of the X-axis (Log Dilution). The correct dilutions used for the serum were from 1/20 to 1/160, which correspond to log dilutions approximately from log 1.3 to log 2.2. Therefore, the X-axis should have been labeled as 1.2 to 2.4, rather than 2.2 to 3.4. We apologize for this oversight and have corrected the figure accordingly to reflect the accurate dilution range. The corrected Figure 10 is listed below:

The authors state that the scientific conclusions are unaffected. This correction was approved by the Academic Editor. The original publication has also been updated.

## Figures and Tables

**Figure 10:**
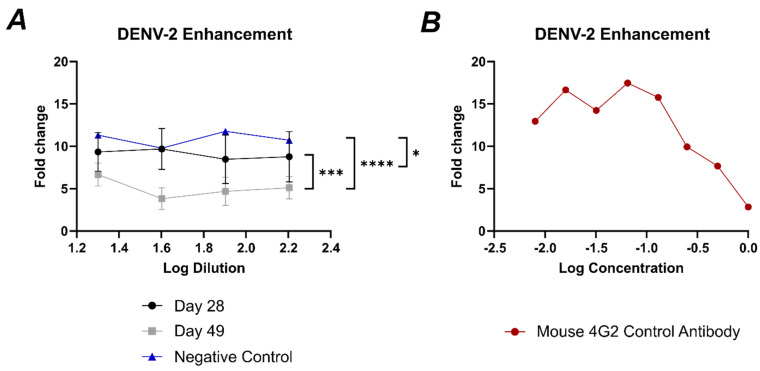
Antibodies induced by vaccination with DV1-AP205/AP205-DV4 do not enhance DENV−2 infection. (**A**) Fold change of DENV-2 infection of the serum-induced by vaccination with DV1-AP205/AP205-DV4. Day 28 is shown in black, and day 49 is shown in grey. Negative Control (naïve serum) is shown in blue. The serum dilution is shown in log values. (**B**) Fold change of DENV-2 infection of mouse 4G2 antibody used as a positive control. Antibody concentration is shown in log values. Statistical analysis (mean ± SEM) using one-way ANOVA. Vaccine group *n* = 6. One representative of two similar experiments is shown. The value of *p* < 0.05 was considered statistically significant (* *p* < 0.05, *** *p* < 0.001, **** *p* < 0.0001).

## References

[1] Rothen D.A., Dutta S.K., Krenger P.S., Vogt A.-C.S., Lieknina I., Sobczak J.M., Osterhaus A.D.M.E., Mohsen M.O., Vogel M., Martina B. (2024). Preclinical Evaluation of Novel Sterically Optimized VLP-Based Vaccines against All Four DENV Serotypes. Vaccines.

