# Supplementary material for: Preclinical Evaluation of Novel Sterically Optimized VLP-Based Vaccines against All Four DENV Serotypes
Source: Vaccines (Basel). 2024 Jul 31;12(8):874. doi: 10.3390/vaccines12080874 (PMC11359203; doi:10.3390/vaccines12080874)
Supplement: Supplementary file 1 [file vaccines-12-00874-s001.zip › vaccines-3113260-supplementary.pdf]

Supplementary Figures

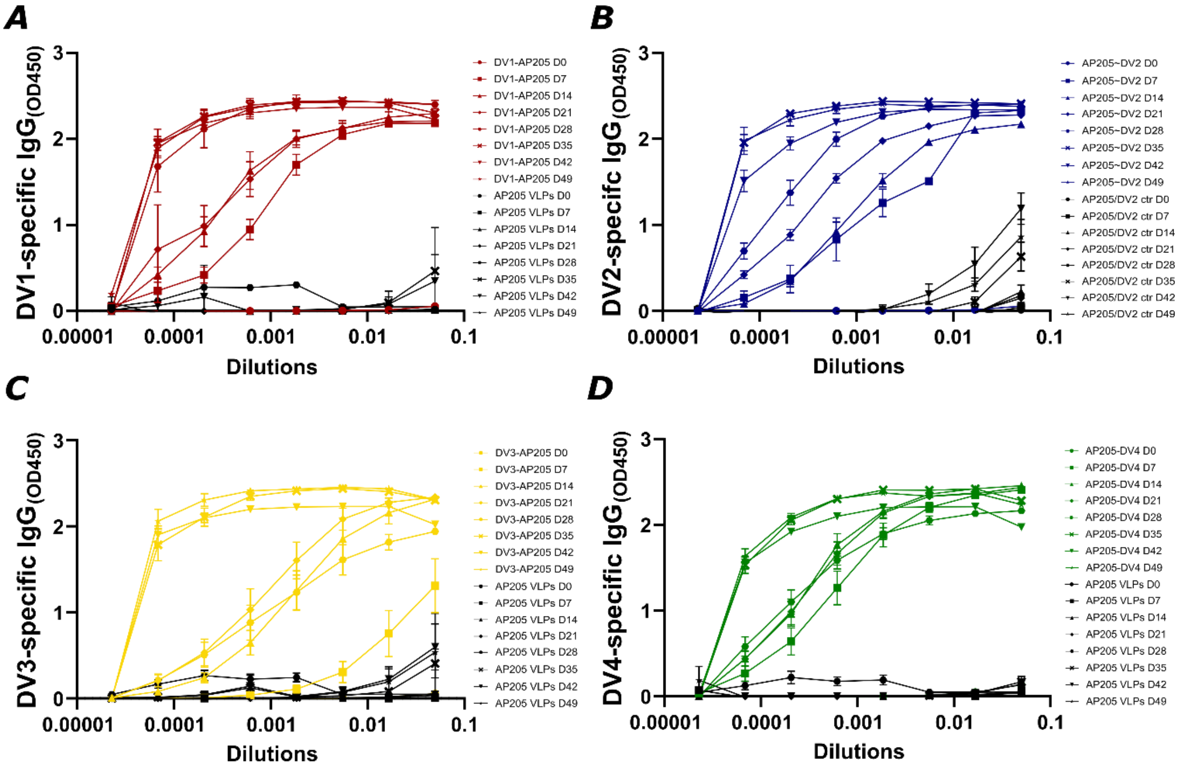

Supplementary Figure S1. Vaccination with the newly developed vaccine candidates induce a strong humoral immune response. ***A)*** DV1-specific IgG titer on days 0, 7, 14, 21, 28, 35, 42 and 49 from mice vaccinated with DV1-AP205 or AP205 VLPs measured by ELISA, OD450 shown. ***B)*** DV2-specific IgG titer on days 0, 7, 14, 21, 28, 35, 42 and 49 from mice vaccinated with AP205~DV2 or AP205 VLPS/DV2 mixture measured by ELISA, OD450 shown. ***C)*** DV3-specific IgG titer on days 0, 7, 14, 21, 28, 35, 42 and 49 from mice vaccinated with DV3-AP205 or AP205 VLPs measured by ELISA, OD450 shown. ***D)*** DV4-specific IgG titer on days 0, 7, 14, 21, 28, 35, 42 and 49 from mice vaccinated with AP205-DV4 or AP205 VLPs measured by ELISA, OD450 shown. Vaccine groups n = 5, control group n = 5. One representative of 3 similar experiments is shown.

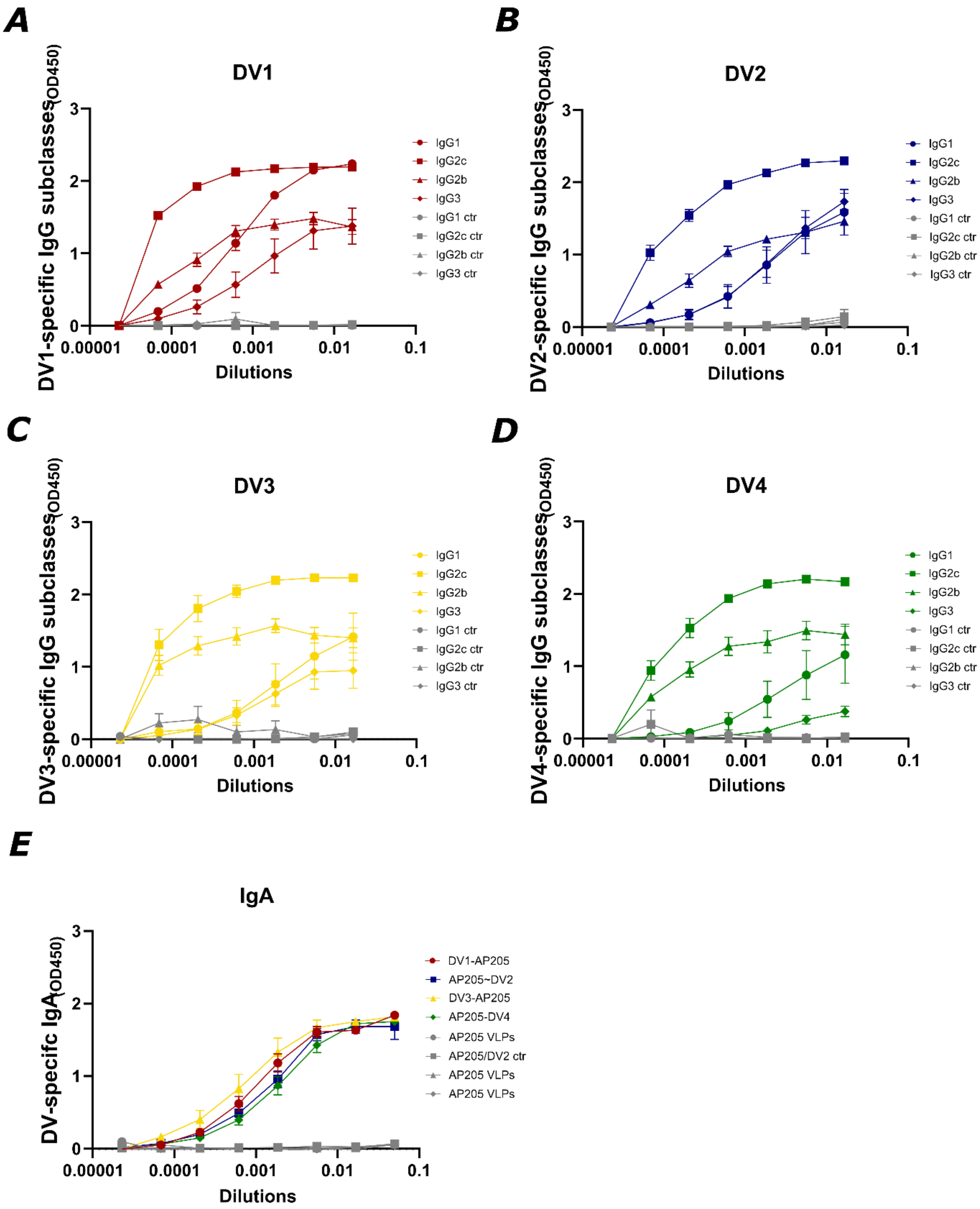

Supplementary Figure S2. Immunization with all four vaccine candidates induces IgG2c and IgG2b dominant Dengue virus Envelope protein domain III specific IgG subclass response and promotes isotype switching to IgA. ***A)*** DV1-specific IgG subclasses titer of day 42 from mice vaccinated with AP205 VLPs as control and DV1-AP205 measured by ELISA, OD450 shown**.** ***B)*** DV2-specific IgG subclasses titer of day 42 from mice vaccinated with AP205 VLPs/DV2 mixture as control and AP205~DV2 measured by ELISA, OD450 shown. ***C)*** DV3-specific IgG subclasses titer of day 42 from mice vaccinated with AP205 VLPs as control and DV3-AP205 measured by ELISA, OD450 shown. ***D)*** DV4-specific IgG subclasses titer of day 42 from mice vaccinated with AP205 VLPs as control and

AP205-DV4 measured by ELISA, OD450 shown. ***E)*** DV-specific IgA titer at day 42 from mice immunized with AP205 VLPs or AP205/DV2 mixture (for DV2-specific) and DV1-AP205, AP205~DV2, DV3-AP05 and AP205-DV4 vaccines measured by ELISA, OD450 shown. Control group n = 5, vaccine groups n = 5. One representative of 2 similar experiments is shown.

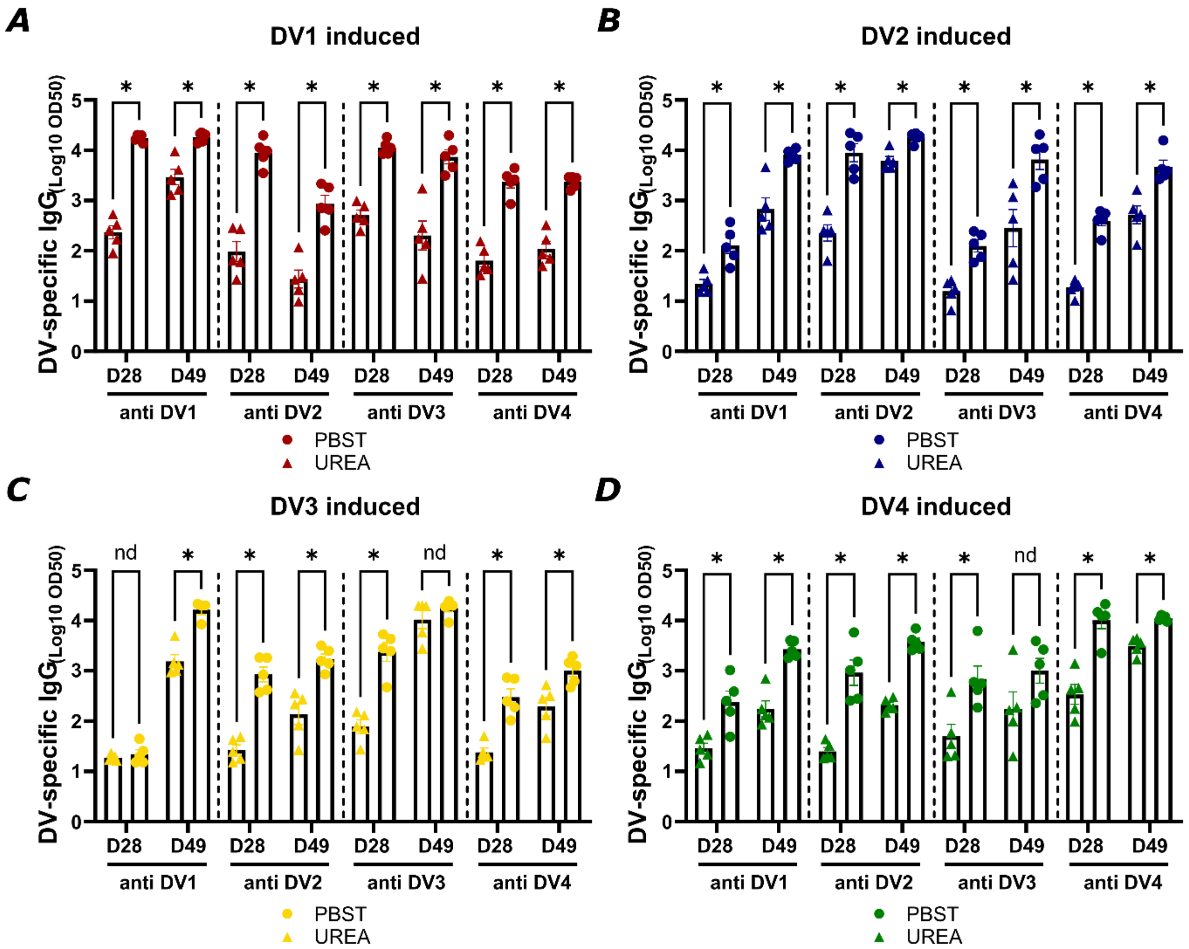

Supplementary Figure S3. Vaccination with DV1-AP205, AP205~DV2, DV3-AP205 and AP205-DV4 elicits high avidity antibodies which are able to recognize the other Dengue serotype EDIII domains due to cross-reactive properties. ***A)*** DV-specific IgG titer of day 28 and 49 from mice vaccinated with DV1-AP205 measured by ELISA, LOG_{10} OD_{50} shown. ***B)*** DV-specific IgG titer of day 28 and 49 from mice vaccinated with AP205~DV2 measured by ELISA, LOG_{10} OD_{50} shown. ***C)*** DV-specific IgG titer of day 28 and 49 from mice vaccinated with DV3-AP205 measured by ELISA, LOG_{10} OD_{50} shown. ***D)*** DV-specific IgG titer of day 28 and 49 from mice vaccinated with AP205-DV4 measured by ELISA, LOG_{10} OD_{50} shown. After serum incubation one plate was treated with PBS +0.05% Tween 20 and the other plate with 7M Urea in PBS+0.05% Tween 20. Statistical analysis (mean ± SEM) using Student`s t-test. Vaccine groups n = 5. One representative of 2 similar experiments is shown. The value of $p < 0.05$ was considered statistically significant (*$p < 0.05$, **$p < 0.01$, ***$p < 0.001$, ****$p < 0.0001$).

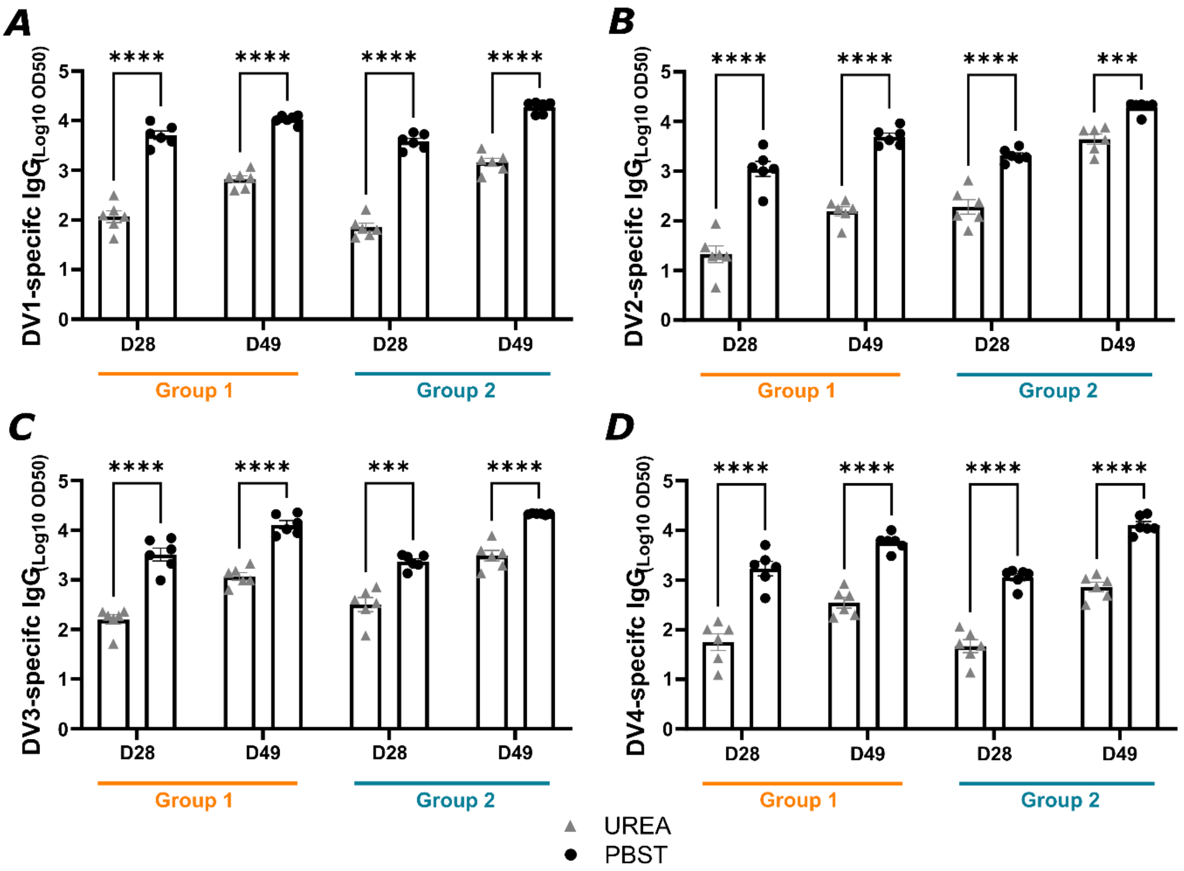

Supplementary Figure S4. Vaccination with all four vaccines compared to vaccination with only two (DV1-AP205/AP205-DV4) tends to increase the humoral immune response with higher avidity. ***A)*** DV1-specific IgG titer of day 49 from group 1 and group 2 measured by ELISA, LOG$_{10}$ OD$_{50}$ shown. ***B)*** DV2-specific IgG titer of day 49 from group 1 and group 2 measured measured by ELISA, LOG$_{10}$ OD$_{50}$ shown. ***C)*** DV-specific IgG titer of day 49 from group 1 and group 2 measured AP205 measured by ELISA, LOG$_{10}$ OD$_{50}$ shown. ***D)*** DV-specific IgG titer of day 49 from group 1 and group 2 measured measured by ELISA, LOG$_{10}$ OD$_{50}$ shown. After serum incubation one plate was treated with PBS+0.05% Tween 20 and the other plate with 7M Urea in PBS+0.05% Tween 20. Statistical analysis (mean ± SEM) using Student`s t-test. Group 1 n = 6, group 2 n = 6. One representative of 2 similar experiments is shown. The value of $p < 0.05$ was considered statistically significant (*$p < 0.05$, **$p < 0.01$, ***$p < 0.001$, ****$p < 0.0001$).

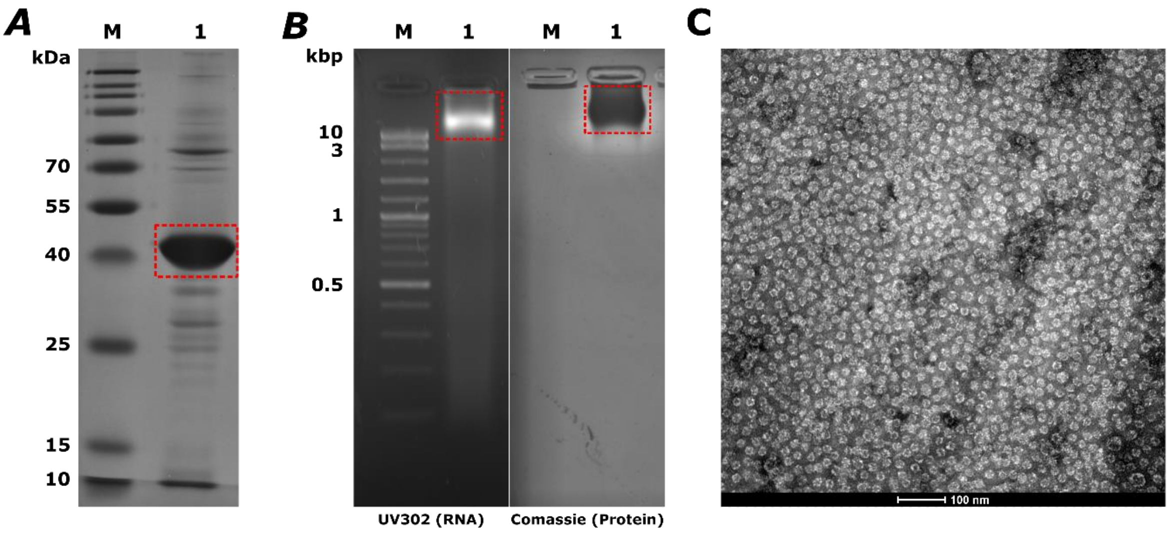

Supplementary Figure S5. Stability of DV1-AP205 after 6 months storage at 4°C. ***A)*** 12% SDS-PAGE for DV1-AP205 after 6 months storage at 4°C. M. Protein marker, 1. DV1-AP205. DV1-AP205 indicated in the red box. Bands were visualized with InstantBlue™ Comassie stain. ***B)*** Agarose gel analysis to visualize the packed RNA in the VLPs and the correlating protein staining with Comassie. M. DNA Ladder, 1. DV1-AP205. DV1-AP205 indicated in the red boxes. ***C)*** Electron microscopy (EM) of DV1-AP205. Scale bar 200nm.

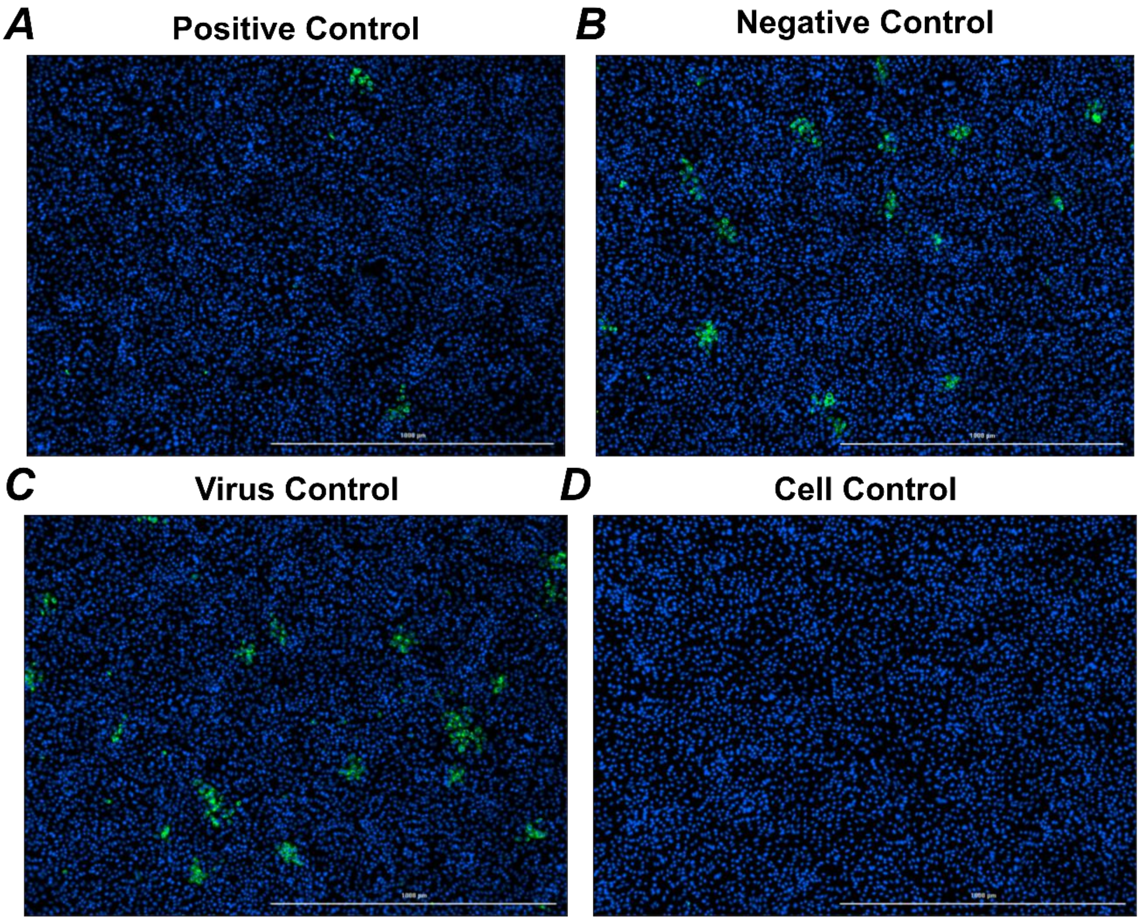

Supplementary Figure S6. Fluorescence Microscopy images of controls on Cells for the Neutralization Assays. ***A)*** Positive Control. ***B)*** Negative Control. ***C)*** Virus Control. ***D)*** Cell Control. DAPI nucleic acid stain is depicted in blue, infected cells are depicted in green. Scale bar 1000 µm.
